# Ribosome profiling reveals multiple roles of SecA in cotranslational protein export

**DOI:** 10.1038/s41467-022-31061-5

**Published:** 2022-06-13

**Authors:** Zikun Zhu, Shuai Wang, Shu-ou Shan

**Affiliations:** 1 Division of Chemistry and Chemical Engineering, California Institute of Technology, Pasadena, CA 91125 USA; 2 Present Address: Department of Molecular and Cellular Physiology, Stanford University, Stanford, CA 94305 USA

**Keywords:** Protein translocation, Biochemical reaction networks, RNA sequencing

## Abstract

SecA, an ATPase known to posttranslationally translocate secretory proteins across the bacterial plasma membrane, also binds ribosomes, but the role of SecA’s ribosome interaction has been unclear. Here, we used a combination of ribosome profiling methods to investigate the cotranslational actions of SecA. Our data reveal the widespread accumulation of large periplasmic loops of inner membrane proteins in the cytoplasm during their cotranslational translocation, which are specifically recognized and resolved by SecA in coordination with the proton motive force (PMF). Furthermore, SecA associates with 25% of secretory proteins with highly hydrophobic signal sequences at an early stage of translation and mediates their cotranslational transport. In contrast, the chaperone trigger factor (TF) delays SecA engagement on secretory proteins with weakly hydrophobic signal sequences, thus enforcing a posttranslational mode of their translocation. Our results elucidate the principles of SecA-driven cotranslational protein translocation and reveal a hierarchical network of protein export pathways in bacteria.

## Abstract (web summary)

Using a combination of ribosome profiling methods, Zhu et al. investigate the principles governing the cotranslational interaction of SecA with nascent proteins and reveal a hierarchical organization of protein export pathways in bacteria.

## Introduction

Generation and maintenance of compartmentalization is essential for the proper functioning of all cells and requires that all newly synthesized proteins be localized to their correct cellular destinations. In bacteria, a quarter of newly synthesized proteins need to be transported onto or across the plasma membrane^1,2^. Inner membrane proteins (IMPs) are cotranslationally recognized by signal recognition particle (SRP), typically via the first transmembrane domain (TMD), and targeted to the SecYEG or YidC translocation machineries at the inner membrane^3–6^. It was generally thought that most secretory proteins are targeted and translocated posttranslationally after they are released from ribosomes. SecA, an evolutionarily conserved and essential ATPase^7^, drives the translocation of preproteins across SecYEG^8–11^. Preprotein translocation can be further assisted by the proton-motive force (PMF)^8,12–14^ and by SecDF, an ancillary complex that associates with SecYEG and ratchets translocating substrates into the periplasm using PMF^15,16^.

Nascent secretory proteins may associate with cytosolic chaperones before reaching the membrane. Some secretory proteins are maintained in the unfolded state by the cytosolic chaperone SecB^17,18^ during their delivery to membrane-bound SecA. The highly abundant ribosome-associated chaperone trigger factor (TF) was also suggested to act as a holdase during the targeting of secretory proteins^19,20^. TF cotranslationally binds numerous secretory proteins beginning at a nascent chain length of ~100 amino acids^21^. Deletion of TF accelerates protein export^22^ and results in more cotranslational translocation of some secretory proteins^21,23^, but the mechanism and the physiological role of TF association with nascent secretory proteins remain unclear.

Recent studies challenge the strictly posttranslational mechanism of SecA-mediated protein targeting and translocation. First, SecA binds the ribosome on uL23 near the nascent polypeptide exit tunnel^24,25^, and mutations that disrupt SecA’s ribosome binding slowed the export of a classic SecB/A substrate, maltose-binding protein (MBP)^24^. In addition, SecA is required for the translocation of multiple IMPs, which must use a cotranslational mechanism of targeting and insertion mediated by SRP^26–30^. Finally, SecA recognizes ribosomes exposing the nascent chain of an IMP, RodZ, with high affinity (*K*_d_ < 1 nM) and is necessary and sufficient for the cotranslational targeting and translocation of RodZ in the absence of SRP^25,31,32^. These observations implicate a role of SecA in cotranslational protein transport.

However, many fundamental questions concerning the cotranslational actions of SecA remain unanswered. The spectrum of nascent proteins that cotranslationally interact with SecA is unclear, as is the precise role of these interactions in protein biogenesis. The timing of SecA-nascent chain association, as well as the determinants of SecA recognition, remain elusive. Moreover, the binding sites of SecA, ribosome, and SecYEG on one another overlap extensively^33–35^, raising questions as to whether and how SecA engages ribosome-translocon complexes cotranslationally. Finally, how SecA coordinates with other ribosome-bound targeting factors and chaperones in bacteria to triage nascent membrane and secretory proteins is not well understood. Here, we decipher the cotranslational actions of SecA by globally profiling SecA-nascent chain interactions and monitoring the targeting of individual substrates in vivo. Our proteome-wide data set identifies the nascent substrate pool of SecA, elucidates the roles of SecA and its coordination with PMF in cotranslational protein translocation, underscores the role of TF in delaying the SecA-mediated transport of a subset of secretory proteins, and reveals a hierarchical network of protein targeting and translocation pathways in bacteria.

## Results

### Cotranslational engagement of SecA with nascent proteins

**Fig. 1:**
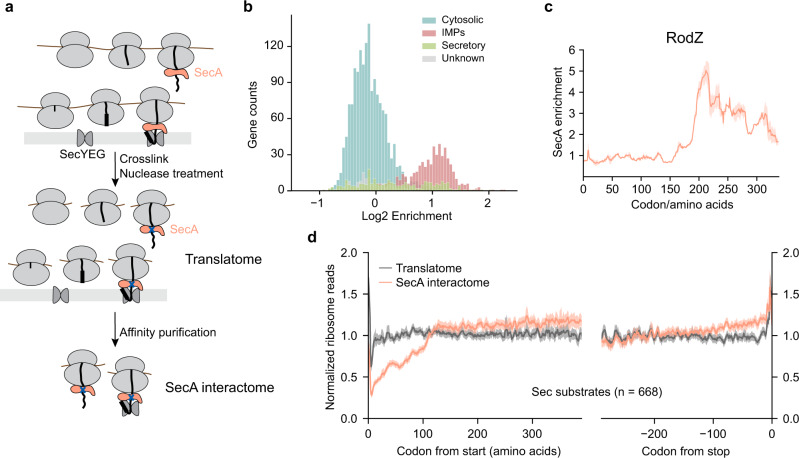
Cotranslational engagement of SecA with RNCs. **a** Schematic of SeRP of SecA. Cells are harvested and cryogenically lysed, followed by crosslinking SecA-RNCs using EDC. Monosomes generated by nuclease digestion of polysomes are isolated by centrifugation through a sucrose cushion. SecA-RNCs are purified from total monosomes via the biotin moiety on Avi-tagged SecA and eluted by thrombin cleavage. Ribosome-protected fragments from the total ribosome population and from SecA-bound RNCs were extracted and sequenced, generating information on the translatome and SecA-interactome. **b** Distribution of SecA enrichment on all genes (*n* = 2562) categorized by protein localization. **c** SecA interaction profile of the RodZ nascent chain. The solid line shows the mean, and the shaded area shows the range of data from two independent biological replicates. **d** Metagene translatome and SecA interactome profiles of Sec substrates aligned to the start and stop codon. Solid lines show the mean values, and shaded areas show the 95% confidence interval (CI).

To understand the principle of SecA-nascent chain interactions, we carried out selective ribosome profiling (SeRP) of SecA in *Escherichia coli* (Fig. 1a). Chromosomal SecA was fused to a C-terminal thrombin-cleavable AviTag, which can be biotinylated by the endogenous biotin ligase, to facilitate the purification of SecA-bound ribosome-nascent chain complexes (RNCs). Very low amounts of ribosomes co-purified with SecA in initial experiments (Supplementary Fig. 1a, lane 3), which was attributed to the transient nature of SecA-RNC interactions. After extensive optimization, we chose to crosslink SecA to nascent chains in the lysate using EDC (1-Ethyl-3-(3-dimethylaminopropyl)carbodiimide), a zero-length crosslinker that couples carboxyl groups with primary amines. Analysis of the elution products from affinity purification showed that addition of EDC significantly increased the yield of ribosomes, whereas no ribosome was detected in parallel purifications from cells with untagged SecA (Supplementary Fig. 1a). Ribosome-protected mRNA fragments from the total ribosome population and from SecA-bound RNCs were then extracted and sequenced, generating information of the translatome and SecA-interactome (Fig. 1a, Supplementary Fig. 1b, c and Supplementary Fig. 2).

To validate that our procedure correctly captured SecA-RNC complexes, we sorted all the detected proteins based on their localization and found that IMPs and a subset of secretory proteins are strongly enriched in the SecA interactome (Fig. 1b and Supplementary Data 1), consistent with a specific role of SecA in protein transport. In addition, the SecA enrichment profile of its model cotranslational substrate, RodZ (Fig. 1c), showed a distinct peak beginning at a nascent chain length of 186 amino acids, indicating a cotranslational engagement by SecA and further supporting the specificity of the affinity purification procedure in faithfully capturing SecA-nascent chain interactions.

We next determined the timing of cotranslational SecA engagement by performing metagene analyses of the total translatome and the SecA interactome on all Sec substrates, including IMPs and secretory proteins, aligned to their start and stop codons (Fig. 1d). The metagene translatome showed a uniform distribution of ribosome footprint read density along the transcript. The read density of the SecA interactome rises gradually during the translation of the first ~120 amino acids, reaches a plateau afterwards, and rises again near the end of translation (Fig. 1d). As described in the sections below, this metagene profile arises from the combination of the distinct timing of SecA interactions with different classes of substrates.

### Fractionation-coupled ribosome profiling reveals the timing of membrane targeting

**Fig. 2:**
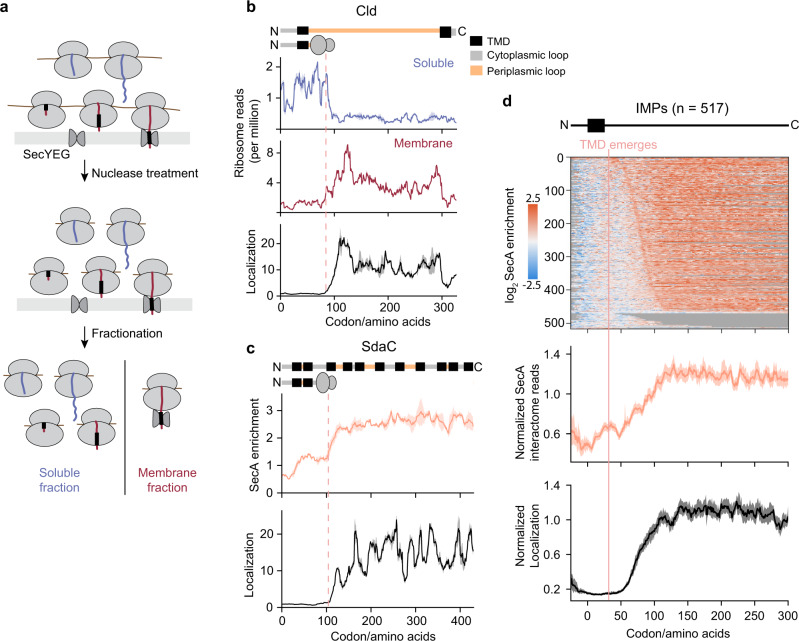
Fractionation-coupled ribosome profiling reveals the timing of cotranslational membrane targeting. **a** Schematic of fractionation-coupled ribosome profiling. **b** Representative ribosome localization profile of an IMP. The localization score at each codon was calculated as the ratio of ribosome-protected reads in the membrane faction relative to those in the soluble fraction. Protein topology is shown above, with TMD in black, cytoplasmic loops in grey, and periplasmic loops in yellow. **c** Representative SecA interaction profile and ribosome localization profile of an IMP. Protein topology is shown above and colored as in **b**. **d** Metagene SecA interactome profile and ribosome localization profile of IMPs aligned to the N-terminus of the first TMD. The heatmap above shows the log_2_ SecA enrichment at each codon of all the IMPs used to derive the metagene SecA interactome profile, sorted by increasing distance from the TMD to the onset of SecA binding. In **b** and **c**, solid lines show the mean values, and shaded areas show the range of data from two independent biological replicates. In **d**, solid lines show the mean values, and shaded areas show the 95% confidence interval (CI).

Considering the important role of SecA in protein translocation, we reasoned that SecA-RNC interaction may be correlated with the timing of nascent chain delivery to the membrane. To test this hypothesis, we developed a protocol, Fractionation-coupled Ribosome Profiling, to monitor cotranslational protein targeting events (Fig. 2a and Supplementary Fig. 3a). In this protocol, we carried out nuclease treatment before cell fractionation, so that only the RNCs stably bound to the membrane via protein-protein or protein-lipid interactions would be retained in the membrane fraction. In contrast, ribosomes on which the nascent chain does not stably engage with components on the membrane would be released into the cytosol after nuclease digestion and captured in the soluble fraction. Ribosome-protected reads from both fractions are then purified, sequenced, and mapped onto protein-coding sequences.

For IMPs, ribosome-protected reads in the soluble fraction declined sharply upon the emergence of the first TMD, with a corresponding rise in the read density of the same protein in the membrane fraction (Fig. 2b). To normalize for local variations in translation speed, we calculated the ratio of membrane reads to soluble reads at each codon, generating the ribosome localization profile for each protein (Fig. 2b). The metagene ribosome localization profile of all 517 IMPs, aligned to the N-terminus of their first TMD, revealed that IMPs on average are targeted to the membrane after their first TMD emerges from the ribosomal exit tunnel (Fig. 2d, lower panel), consistent with previous reports that SRP recognizes the first TMD of IMPs for targeting^4^. We also observed delayed targeting in the ribosome localization profiles of IMPs whose first TMD is skipped by SRP, which confirmed their SRP-dependent targeting (Supplementary Fig. 3b). In contrast, the ribosome localization profiles of cytosolic proteins remained unchanged throughout translation (Supplementary Fig. 3c, d).

**Fig. 3:**
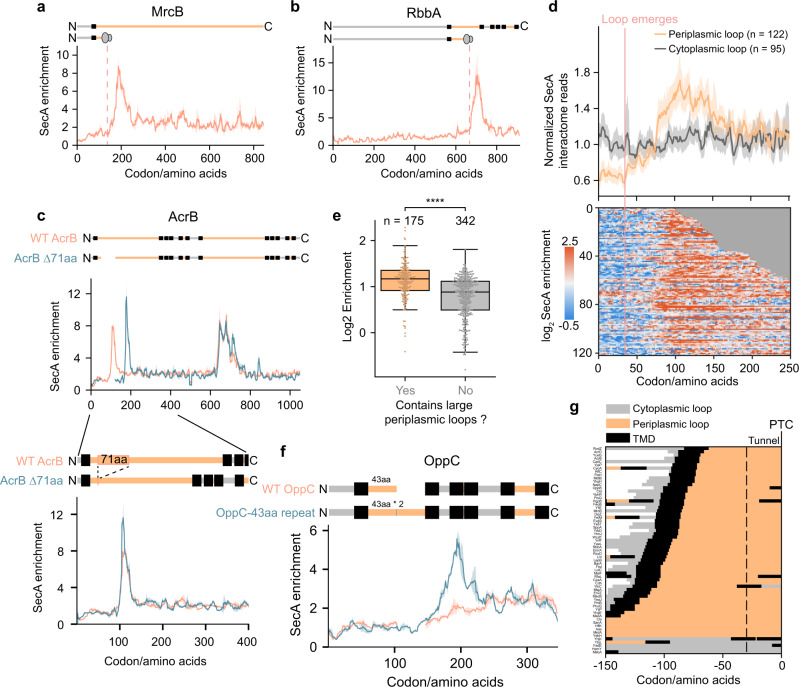
SecA cotranslationally translocates large periplasmic loops of IMPs. **a**, **b** Representative SecA interaction profiles of IMPs with large periplasmic loops. Protein topology is shown above and colored as in Fig. 2b. **c** Upper panel, comparison of SecA interaction profile of WT AcrB and AcrB-∆71aa. The topology of the protein is shown above and colored as in Fig. 2b. The deleted region was skipped in the indicated protein topology and SecA interaction profile of AcrB-∆71aa so that SecA enrichment at each codon corresponds to the same protein sequence between WT AcrB and AcrB-∆71aa. Lower panel, zoom in of the first 400 codons. SecA enrichment is plotted as a function of ribosome position relative to the start codon for each protein. aa, amino acids. **d** Metagene SecA interactome profile of periplasmic and cytoplasmic loops larger than 100 amino acids aligned to their N-terminus. The heatmap below shows the log_2_ SecA enrichment at each codon for all the periplasmic loops used to derive the metagene SecA interactome profile, sorted by the length of each periplasmic loop. **e** Comparison of gene-level enrichment between proteins with and without a large periplasmic loop. **f** Comparison of SecA interaction profile of WT OppC and OppC-43aa repeat, which contains two tandem copies of the 43aa periplasmic loop in WT OppC. Protein topology is shown above and colored as in Fig. 2b. The inserted region (43 amino acids) was skipped in the protein topology and SecA interaction profile of WT OppC so that the SecA enrichment at each codon corresponds to the same protein sequence between WT OppC and OppC-43aa repeat. aa, amino acids. **g** Topology of translated nascent chains of strong SecA interactors at the onset of SecA binding. The nascent chains are aligned to their C-terminus (position 0, at PTC) and the residues to the left of the dashed line are exposed outside the ribosomal tunnel exit. In **a**–**c** and **f**, solid lines show the mean values, and shaded areas show the range of data from two independent biological replicates. In **d**, solid lines show the mean values, and shaded areas show the 95% CI. In **e**, the centre lines show the median, the boxes show the range of data between the upper and lower quartiles, and the whiskers indicate 1.5x the interquartile range. *P* = 8.326e^−17^, from two-sided Wilcoxon rank-sum test.

We next compared the onset of SecA enrichment with the timing of membrane association for all IMPs (Fig. 2d). Except for a few IMPs that are too short to be cotranslationally targeted, 91% of all 517 IMPs detected display a gradual rise in SecA enrichment after the emergence of their first TMD, which coincides with their membrane association (Fig. 2d and Supplementary Fig. 3e). In the interaction profiles of individual IMPs, SecA binding increases concurrently with their membrane association but showed remarkably different patterns afterwards. On most IMPs, SecA association quickly plateaus at SecA enrichment of 2-3 fold and continues until the end of translation (Fig. 2c and Supplementary Fig. 3e). On a subset of IMPs, however, we observed strong SecA binding peaks that rise above and then return to this plateau (Fig. 3a–c and described in the next section). Thus, the low and persistent level of SecA enrichment, which displays no specificity in the length or identity of the nascent chain, may represent transient interactions between membrane-bound SecA and the translocating RNCs until a specific and stable interaction is established.

### SecA cotranslationally translocates large periplasmic loops of inner membrane proteins

We further investigated the strong SecA binding peaks on IMPs, which appeared when part of a large periplasmic loop has emerged from the ribosomal exit tunnel (Fig. 3a–c and Supplementary Fig. 4a, b). These peaks typically persist for more than 30 codons and are independent of the number of TMDs in the protein (Fig. 3a–c and Supplementary Fig. 4a, b). Interestingly, for proteins with more than one large periplasmic loop, strong SecA interaction peaks could be observed multiple times, with binding peaks correlating with a partially exposed periplasmic loop (Fig. 3c). These observations agree with previous reports showing that the translocation of IMPs containing large periplasmic loops requires SecA^26–30^, suggesting that SecA stably engages RNC-SecYEG complexes during the cotranslational translocation of large periplasmic loops.

To determine the timing of SecA engagement on the periplasmic loops, we generated a metagene SecA interactome profile for all the predicted periplasmic loops longer than 100 amino acids aligned to their N-terminus (Fig. 3d). Loops N-terminal to the first TMD, which are not targeted to the membrane during their synthesis, were excluded from this analysis. SecA on average initiates binding when the N-terminus of a large periplasmic loop reaches a distance of 75 amino acids from the peptidyl transferase center (PTC), and maximal binding is reached at a distance of 110 amino acids from the PTC (Fig. 3d). Assuming that the C-terminal ~30 amino acids of the nascent chain are buried in the ribosomal exit tunnel, the emergence of at least 45 amino acids of the loop from the ribosome is required for strong SecA engagement. Consistently, IMPs containing a periplasmic loop longer than 45 amino acids are significantly enriched in the SecA interactome in a gene-level comparison (Fig. 3e).

To test whether the length or specific sequence features of a periplasmic loop determines SecA engagement, we studied how mutations introduced in the periplasmic loops of two proteins, OppC and AcrB, impact SecA binding (Fig. 3c, f). WT OppC has a periplasmic loop of 43 amino acids that does not cotranslationally recruit SecA (Fig. 3f). However, introduction of a tandem repeat of this stretch of 43 amino acids in OppC-43aa repeat, which only increased the length of the periplasmic loop, led to the appearance of a strong SecA binding peak after ~60 amino acids of the altered loop have emerged from the ribosome (Fig. 3f), in agreement with the timing of SecA engagement observed with the metagene SecA interactome (Fig. 3d). The second protein, AcrB, contains two large periplasmic loops that both strongly engage SecA (Fig. 3c). The first SecA binding peak ends at residue 105 of the first periplasmic loop. Considering that over 20 amino acids at the N-terminus of the loop are inserted into SecYEG for a TMD in type II topology^34^, and at least 30 amino acids^36^ at the C-terminus of the loop are still in the ribosomal exit tunnel, we reasoned that the emergence of residues 20–75 in this loop determined SecA association. If SecA engagement is sequence-dependent, it would be abolished with AcrB-Δ71aa, in which residues 20–90 of this loop is deleted. Instead, the truncation led to a new SecA binding peak at residues 70–105 of the new periplasmic loop (Fig. 3c), at a protein sequence that showed no SecA binding in WT AcrB (Fig. 3c). These data indicate that high affinity SecA binding to RNC-SecYEG complexes is determined by the length, rather than specific amino acid sequence, of periplasmic loops of IMPs.

We next explored if SecA also interacts with other segments of nascent IMPs besides large periplasmic loops (e.g., TMDs of some proteins). We developed a peak detection algorithm to identify SecA peaks with at least 12 consecutive codons above a threshold of 3.5-fold enrichment. The stringent criteria ensures that only the strongest SecA interactors were detected and excludes the baseline levels of nonspecific SecA enrichment. Among the 63 substrates detected by this method, 58 of them expose a large periplasmic loop outside the ribosome at the onset of SecA binding (Fig. 3g). A closer inspection of the other 5 proteins, which lacks large periplasmic loops, reveals only a higher baseline of SecA enrichment (Supplementary Fig. 4d). At the metagene level, the onset of strong SecA binding occurs after the protein is completely targeted to the membrane (Supplementary Fig. 4e), reinforcing SecA’s involvement in protein translocation. Together, these findings indicate that driving the translocation of large periplasmic loops is the primary role of SecA in the cotranslational insertion of IMPs.

### Distinct modes of SecA engagement on nascent secretory proteins

**Fig. 4:**
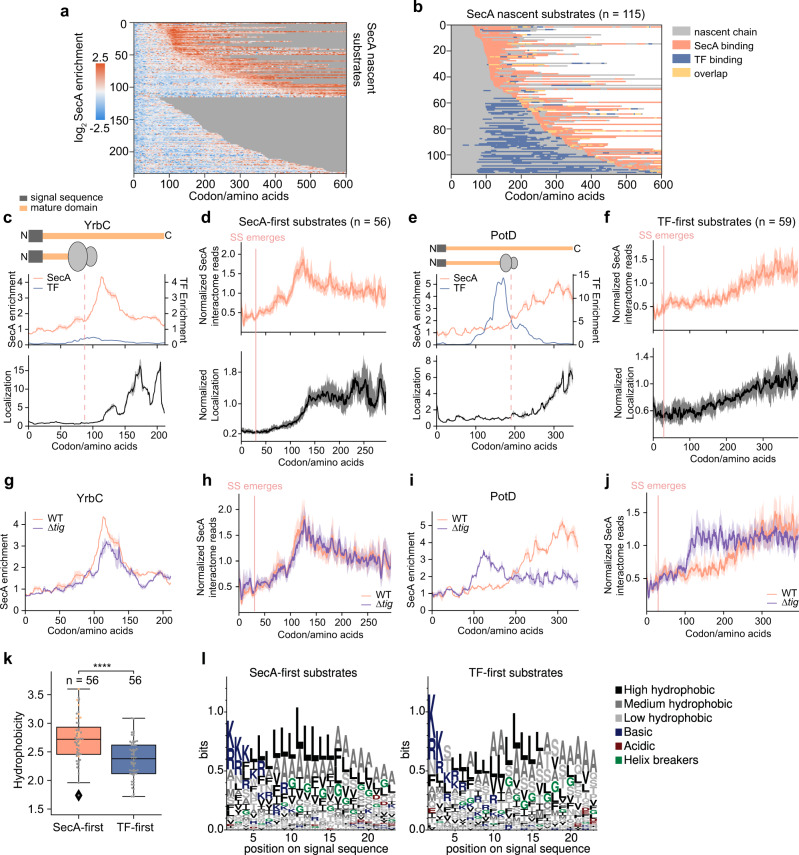
SecA engagement on nascent secretory proteins is delayed by trigger factor. **a** Heatmap of log_2_ SecA enrichment at each codon of all secretory proteins. **b** Heatmap of SecA and TF binding^21^ on SecA-interacting nascent secretory proteins, sorted by increasing distance from the start codon to the onset of SecA binding. **c**, **e** Representative SecA and TF^21^ interaction profiles and ribosome localization profiles of a SecA-first (**c**) and a TF-first (**e**) substrate. Protein topology is shown above, with signal sequence in dark grey and mature domain in yellow. **d**, **f** Metagene SecA interactome profile and ribosome localization profile of SecA-first (**d**) and TF-first (**f**) substrates aligned to the N-terminus of signal sequences. **g**, **i** Representative SecA interaction profiles of a SecA-first (**g**) and a TF-first (**i**) substrate. Protein topology is shown above and colored as in **c**. **h**, **j** Metagene SecA interactome profile of SecA-first (**h**) and TF-first (**j**) substrates aligned to the N-terminus of signal sequences. **k**, Hydrophobicity of the signal sequences of SecA-first and TF-first substrates. Yellow dots indicate substrates that also engage with SRP. The signal sequences of Ppk, SpeA and HybC cannot be faithfully predicted and are excluded from the analysis. Source data are provided in the “Source Data” file. **l**, WebLogo representations of the amino acid compositions of the signal sequences of SecA-first and TF-first substrates aligned to the second amino acid. In **c**, **e**, **g**, **i**, solid lines show the mean values, and shaded areas show the range of data from two independent biological replicates. In **d**, **f**, **h**, **j**, solid lines show the mean values, and shaded areas show the 95% CI. In **k**, the centre lines show the median, the boxes show the range between the upper and lower quartiles of data, and the whiskers indicate 1.5x the interquartile range. *P* = 3.716e^−6^, from two-sided Wilcoxon rank-sum test.

We next investigated the interaction of SecA with nascent secretory proteins. To identify substrates of SecA among nascent secretory proteins, we used the peak detection algorithm to scan for regions with at least 12 codons above a threshold of 1.7-fold SecA enrichment. We identified 115 SecA interactors out of 233 reliably detected secretory proteins (Fig. 4a), indicating that approximately half of the nascent secretory proteome contact SecA before they are completely synthesized and released from the ribosome. Since the chaperone TF also interacts with nascent secretory proteins, we overlaid the TF binding peaks^21^ on the identified SecA substrates (Fig. 4b). SecA and TF share many substrates but seldom bind simultaneously on RNCs. Two classes of SecA substrates were then sorted based on which factor engages first.

Among the 115 nascent secretory substrates of SecA, 56 contact SecA before TF or do not engage TF throughout translation (referred to as SecA-first substrates; Fig. 4b, c and Supplementary Fig. 5a, b). The metagene SecA interactome profile of these SecA-first substrates showed that SecA binding begins when the nascent chain is ~100 amino acids long. SecA engagement persists for ~50 amino acids (Fig. 4d) and declines afterwards, long before protein synthesis is finished, which is analogous to the SecA interaction on the large periplasmic loops of translocating IMPs. Different from the IMPs (Supplementary Fig. 4e), however, there is no lag between the membrane association of these secretory proteins and the onset of SecA binding peak. Instead, SecA engagement precedes their membrane association (Fig. 4c, d and Supplementary Fig. 5a, b), suggesting a role of SecA in both the membrane targeting and initial translocation of these secretory proteins. TF seldom binds these substrates after SecA engagement (Fig. 4b). Collectively, these data suggest a SecA-mediated cotranslational export pathway utilized by ~25% of secretory proteins.

The other 59 SecA-interacting nascent secretory proteins first contact TF at a length of ~100 amino acids, and SecA does not bind these proteins until after TF dissociation (referred to as TF-first substrates; Fig. 4b, e and Supplementary Fig. 5c, d). The interaction of SecA with TF-first substrates rises with increasing nascent chain length and lasts until the end of translation at both the metagene level and for individual proteins (Fig. 4b, e, f and Supplementary Fig. 5c, d). Considering the ability of SecA to bind secretory proteins post-translationally, SecA may continue to engage these substrates after they complete translation and are released from the ribosome. The membrane targeting of these TF-first substrates was also relatively late, occurring near the end of translation (Fig. 4e, f and Supplementary Fig. 5c, d). Thus, the observations on TF-first substrates may reflect a temporally cotranslational but mechanistically posttranslational mechanism of export^37^ mediated by SecA.

### TF delays SecA engagement on nascent secretory proteins

To further understand the molecular interplay between SecA and TF, we analyzed the SecA interactome in cells lacking the *tig* gene encoding TF. The timing of SecA engagement on SecA-first substrates was unaffected by *tig* deletion, although the enrichment was slightly lower for individual genes (Fig. 4g, h and Supplementary Fig. 6a, b). In contrast, we observed much earlier SecA interactions with TF-first substrates in the *∆tig* strain, starting at a nascent chain length of ~100 amino acids (Fig. 4i, j and Supplementary Fig. 6c, d). In addition, deletion of TF resulted in the cotranslational engagement of SecA with secretory proteins that, in WT cells, interacted with TF throughout translation and did not cotranslationally bind SecA (Supplementary Fig. 6e–h). These results suggest that SecA by itself does not distinguish and sort secretory proteins into co- or post- translational pathways. Instead, TF selectively interacts with a subset of secretory proteins and delays their early engagement with SecA, thereby enforcing a posttranslational mode of their export.

To explore what determines the early recognition of TF versus SecA on nascent secretory proteins, we compared the Kyte-Doolittle hydrophobicity score of the signal sequences of the two classes of SecA substrates (Fig. 4k), since only the signal sequence and the following ~50 amino acids are exposed outside the ribosome at the onset of SecA or TF binding. The signal sequences of TF-first SecA substrates are significantly less hydrophobic than those of the SecA-first substrates. Furthermore, thirteen of the SecA-first substrates recruit SRP cotranslationally^4^, consistent with the higher hydrophobicity of their signal sequences (Supplementary Fig. 6i). We next performed a position-wise analysis of sequence logos for the signal sequences^38^ and found that SecA-first substrates are more enriched in hydrophobic residues, especially leucine (Fig. 4l). The hydrophobicity of signal sequences of non-SecA substrates, most of which are recognized by TF, are similar to that of TF-first SecA substrates (Fig. 4l and Supplementary Fig. 6j, k). Thus, hydrophobicity of the signal sequence is one of the factors that dictates differential recognition of preproteins by TF versus SecA. TF preferentially recognizes secretory proteins with less hydrophobic signal sequences and delays their interaction with SecA.

We further asked what feature necessitates the use of a cotranslational pathway of export for SecA-first substrates. Considering that the narrow SecYEG pore only allows the passage of unfolded proteins^39,40^, one possibility is that cotranslational translocation ensures the successful export of secretory proteins that fold rapidly and stably, which may otherwise block the translocon in a posttranslational export pathway^41,42^. To test this hypothesis, we calculated the absolute contact order for all SecA-interacting nascent substrates. The absolute contact order is a measure of the total distance between residues that form native contacts in the folded protein and was shown to accurately predict protein folding rates^43^. Interestingly, SecA-first substrates have significantly lower absolute contact order compared to TF-first substrates (Supplementary Fig. 6l), indicating their propensity to fold more quickly. This may explain, in part, the cotranslational engagement of these nascent proteins with translocation machineries.

### Impact of SecB on SecA-nascent chain interaction

SecB is an interaction partner of SecA during the posttranslational export of preproteins and has the ability to engage nascent polypeptides^44,45^. We therefore explored whether SecB impacts the cotranslational interaction of SecA with nascent proteins. To this end, we performed SeRP in two mutant *E. coli* strains: *∆secB*, in which the *secB* gene is deleted; and *secA∆ZnBD*, in which we chromosomally deleted the zinc binding domain (ZnBD; aa 885-896) of SecA required for SecA-SecB binding^46^ to minimize cell stress responses that may be triggered by SecB deletion^47^.

**Fig. 5:**
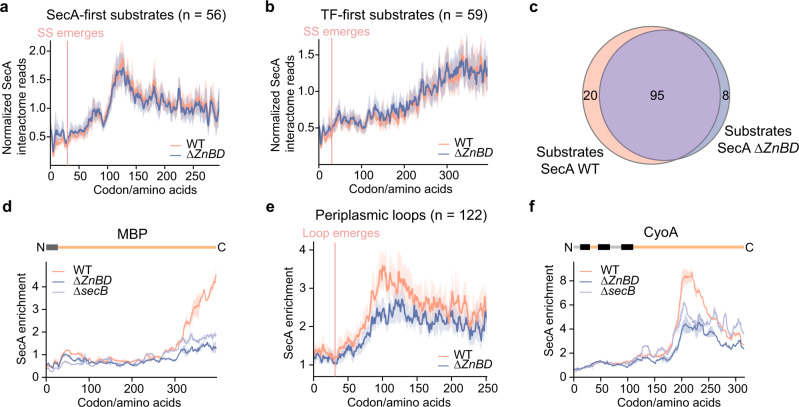
Impact of SecB on cotranslational SecA interactions. **a**, **b** Metagene SecA interactome profile of SecA-first (**a**) and TF-first (**b**) substrates aligned to the N-terminus of signal sequences. Solid lines show the mean values, and shaded areas show the 95% CI. **c** Venn diagrams showing the overlap of nascent secretory substrates of SecA and SecA∆ZnBD. **d**, **f** Representative SecA interaction profiles of a secretory protein (**d**) and an IMP (**f**) on which SecA binding is compromised. Protein topology is shown above and colored as in Figs. 2b and 4c. Solid lines show the mean values, and shaded areas show the range of data from two independent biological replicates. **e** Metagene SecA enrichment of large periplasmic loops aligned to their N-terminus. The median values of SecA enrichment in WT strain and in ∆ZnBD strain are compared. Shaded areas show the 95% CI.

Unlike the observations with the *∆tig* strain, the timing of SecA engagement with nascent secretory proteins was unaffected in both the *∆secB* and *secA∆ZnBD* strains (Fig. 5a, b and Supplementary Fig. 7a, b), indicating that SecA binding on most nascent secretory proteins is independent of SecB. Furthermore, the peak detection analyses showed a heavy overlap of nascent secretory protein substrates of WT SecA and SecA∆ZnBD, with only 20 proteins that interacted exclusively with WT SecA and 8 that interacted exclusively with SecA∆ZnBD (Fig. 5c). A closer inspection of these 28 proteins showed that only SecA engagement on nascent MBP, a classic SecB substrate, is clearly dependent on the SecA-SecB interaction (Fig. 5d). The other 27 proteins have a modest difference in the enrichment score of the SecA binding peaks, such that they fell below or rose above the criteria in our peak detection algorithm (Supplementary Fig. 7c). A comparison of the gene-level enrichment of the overlapping substrates further identified 7 proteins whose interaction with SecA were severely impaired by the ∆ZnBD mutation (Supplementary Fig. 7d, e). These defects were also observed in SecB deletion cells (Fig. 5d and Supplementary Fig. 7c–e). Thus, our results indicate that SecB may facilitate but is not strictly required for SecA recognition on nascent secretory proteins.

Notably, disruption of the SecA-SecB interaction or deletion of SecB led to a reduction in the cotranslational binding of SecA on the large periplasmic loops of IMPs (Fig. 5e, f and Supplementary Fig. 7f). This reduction was not observed with SecA interaction on nascent secretory proteins (Supplementary Fig. 7g, h), indicating that it did not arise from experimental variations. These results suggest that, although SecA can cotranslationally engage with the large periplasmic loops of IMPs in the absence of SecB, SecB helps stabilize the formation of high affinity RNC-SecY-SecA complexes during translocation.

### Proper cotranslational SecA interaction requires the proton motive force

An unexpected feature of cotranslational SecA engagement is that most SecA binding events peak within ~50 amino acids after its onset, and decline long before the periplasmic loop is completely translated. These observations suggest that other driving forces may also be involved in protein translocation. One candidate is PMF, which stimulates the translocation of multiple model substrate proteins^12–14^. Disruption of PMF also affects the localization of SecA within the cell^48^. We therefore analyzed the SecA interactome in cells treated with carbonyl cyanide m-chlorophenyl hydrazone (CCCP), which dissipates the PMF.

**Fig. 6:**
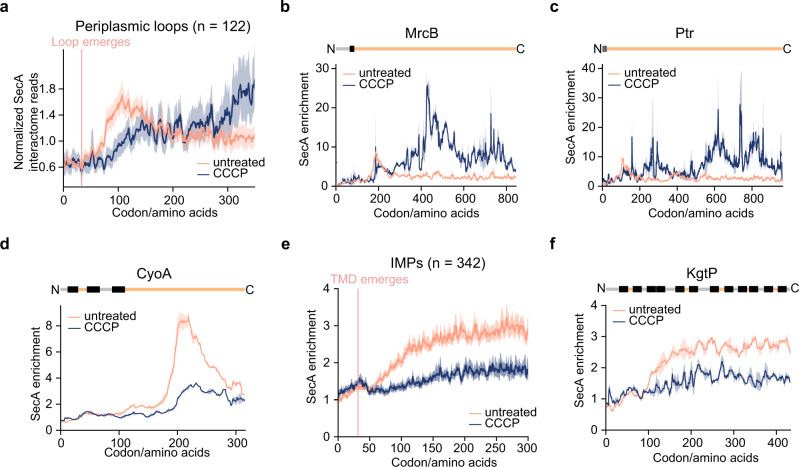
PMF regulates cotranslational SecA interactions. **a** Metagene SecA interactome profile of periplasmic loops larger than 100 amino acids aligned to their N-terminus. Solid lines show the mean values, and shaded areas show the 95% CI. **b**, **c** Representative SecA interaction profile of an IMP with a large periplasmic loop (**b**) and a secretory protein (**c**) that showed persistent SecA association upon CCCP treatment. Protein topology is shown above and colored as in Figs. 2b and 4c. **d** Representative SecA interaction profile of an IMP with a large periplasmic loop that showed significantly reduced SecA association upon CCCP treatment. Protein topology is shown above and colored as in Fig. 2b. **e** Metagene SecA enrichment of IMPs with no periplasmic loops longer than 45 amino acids aligned to the N-terminus of their first TMD. The median SecA enrichment values in CCCP-treated and untreated cells are compared. Shaded areas show the 95% CI. **f** Representative SecA interaction profile of an IMP without a large periplasmic loop in untreated and CCCP-treated cells. Protein topology is shown above and colored as in Fig. 2b. In **b**, **c**, **d**, **f**, solid lines show the mean values, and shaded areas show the range of data from two independent biological replicates.

We first used the peak detection algorithm to identify the strongest SecA-nascent IMP interactions upon CCCP treatment. SecA engagement on all except two substrates started after the emergence of a large periplasmic loop (Supplementary Fig 7a), suggesting that SecA retains its role in translocating large periplasmic loops under conditions of PMF dissipation. However, the metagene SecA interactome of large periplasmic loops of IMPs aligned to their N-terminus showed that PMF dissipation profoundly alters the timing of SecA engagement with RNCs (Fig. 6a).

Inspection of the SecA interaction profiles of individual proteins revealed two distinct effects of PMF dissipation. Half of the 122 large periplasmic loops displayed prolonged SecA engagement (Fig. 6b and Supplementary Fig. 8b). Sustained SecA binding was also observed on some of the nascent secretory proteins upon CCCP treatment (Fig. 6c and Supplementary Fig. 8c). This is consistent with the results of a recent single molecule imaging study, which showed repeated SecA localization at specific membrane loci upon the dissipation of PMF^48^. In contrast, the other half of large periplasmic loops showed significantly reduced SecA engagement or even lost SecA interaction (Fig. 6d and Supplementary Fig. 8d). In addition, SecA enrichment on IMPs without a large periplasmic loop, which represents transient encounters of SecA as it diffuses on the inner membrane, is also significantly reduced in the absence of PMF (Fig. 6e, f). These results are in agreement with the reduced SecA diffusion on the plasma membrane upon CCCP treatment^48^. To test if this loss of SecA engagement is due to a failure of the membrane targeting of substrate proteins, we carried out fractionation-coupled ribosome profiling in CCCP-treated cells. Comparison of the ribosome localization profiles in CCCP-treated and untreated cells revealed that cotranslational protein targeting is unaffected by PMF dissipation (Supplementary Fig. 8e–h), excluding this possibility. Together, these results show that the proper timing and specificity of SecA’s cotranslational interaction with IMPs and secretory proteins require an intact PMF.

## Discussion

Despite recent progress in elucidating the molecular details of SecA-nascent chain interactions on the ribosome, the role of SecA’s cotranslational actions in vivo and its relationship with other protein export pathways in bacteria remain elusive. Here, we used SeRP and fractionation-coupled ribosome profiling to address these questions. Our approaches generated proteome-wide cotranslational targeting and SecA interaction profiles at near-codon resolution in *Escherichia coli*, which allows us to define the nascent substrate pool of SecA in vivo and identify determinants of the timing and specificity of these interactions. Our results uncover the role of SecA in resolving topological problems during the cotranslational translocation of large periplasmic loops. Moreover, comparison of the SeRP profiles of SecA with those of SRP and TF, coupled with genetic perturbations, reveal a hierarchical network of chaperones/targeting factors that ensure robust protein transport in bacteria.

At the gene level, the SecA interactome is strongly enriched in IMPs (Fig. 1b), consistent with a microarray analysis of mRNAs copurified with SecA^49^. However, the modest SecA enrichment level along the transcripts of most IMPs and the lack of specificity of these interactions may reflect SecA in a scanning mode, in which it diffuses along the membrane to sample RNC-SecYEG complexes. In agreement with this interpretation, a recent single molecule imaging study showed that SecA is predominantly localized at and rapidly diffuses on the plasma membrane^48^. Given the heavy overlap of SecA and ribosome binding sites on SecYEG^33–35,50^, SecA interaction with SecYEG or RNC is likely transient and quickly displaced by the RNC-SecYEG interaction. Nevertheless, this scanning mode might enable SecA to sense its substrates on SecYEG in a timely manner, allowing it to initiate more stable engagement with specific substrates.

Notably, SecA strongly and specifically engages translocating periplasmic loops of IMPs after ~45 amino acids of the loop emerge from the ribosome (Fig. 3). Crystal structure of a translationally stalled RNC-SecY complex showed that the mature domain of DsbA forms a loop on the cytosolic surface of SecY, rather than entering the channel directly, during initiation of cotranslational translocation^34^. Our data agree with this structural observation and suggest that, for proteins with large periplasmic loops, cytosolic accumulation and exposure of the periplasmic sequence is a ubiquitous phenomenon during their cotranslational translocation in vivo, which could finally disrupt the ribosome-SecYEG junction. SecA specifically recognizes these species as soon as the topological problems emerge, and likely uses its ATPase cycle to drive the translocation of the periplasmic loops across SecYEG^15,51,52^. This provides a molecular model to explain why IMPs with large periplasmic loops tend to display a strong dependence on SecA for translocation^26–30^.

Interestingly, most of these strong SecA interactions persist for only 50-100 amino acids and declines long before the periplasmic loop is completely translated (Fig. 3a–d), suggesting that SecA is only responsible for initial translocation. One possibility is that SecA-driven translocation across SecYEG is faster than translation elongation^2,53^, which quickly resolves the cytosolic accumulation of the periplasmic region. The ribosome is then able to gain close approach to SecYEG to recover the ribosome-translocon junction, outcompeting SecA during this process. Subsequent translocation could be driven by other forces including PMF^12–14^, PMF-driven pulling by SecD/F^16^, folding of the periplasmic domains^54,55^, or their binding by periplasmic chaperones^56,57^. Efficient continuous translocation prevents further accumulation of the periplasmic loop in the cytosol, thus alleviating the need for SecA during later stages of translocation. In support of this hypothesis, dissipation of the PMF leads to prolonged SecA association with large periplasmic loops of a subset of IMPs (Fig. 6a, b) which might reflect repeated attempts of SecA to reinitiate translocation in the absence of a driving force for continued translocation. Together, our findings reveal the role of SecA in resolving topological problems encountered by SecYEG during the cotranslational translocation of proteins with large periplasmic loops.

SecA also cotranslationally engages ~50% of the secretory proteins in two distinct modes depending on their signal sequence (Fig. 4). For secretory proteins harboring highly hydrophobic signal sequences, SecA association occurs early, long before protein synthesis is finished. Their membrane association occurs following SecA engagement, suggesting that SecA can cotranslationally target and translocate these substrates (Fig. 4c, d, k). An example is DsbA, a periplasmic protein with a hydrophobic signal sequence known to drive cotranslational protein export^42^. DsbA was assumed to be an SRP substrate^42^ but did not associate with SRP in a ribosome profiling study^4^. The early cotranslational association of DsbA with SecA (Supplementary Fig. 5b) resolves these inconsistencies and explains why DsbA export showed a stronger dependence on SecA than on SRP^42^. In addition, the rapid and stable folding of the mature domain of DsbA in the cytosol was suggested to necessitate the cotranslational mechanism of its export^42,58^. Here we found that this feature is enriched among SecA-first substrates, suggesting that signal sequences co-evolved with the biophysical properties of the nascent protein to optimize their export efficiency. Taken together, our results suggest that ~25% of secretory proteins in bacteria can use a SecA-mediated cotranslational export pathway.

In contrast, secretory proteins with weakly hydrophobic signal sequences associate with TF at a nascent chain length of ~100 amino acids and display a gradual rise in SecA enrichment and membrane association only after the dissociation of TF (Fig. 4e, f, k). We speculate that the translocation of these proteins is only temporally cotranslational but not mechanistically coupled to translation elongation^59,60^, which is distinct from the SRP- and SecA-mediated cotranslational transport discussed earlier. However, disruption of SecA-ribosome interaction in vivo leads to accumulation of the precursor form of a TF-first substrate, MBP (Fig. 5d)^24^, suggesting that although the mechanism of their translocation is analogous to a posttranslational pathway, the ribosome interaction of SecA facilitates their export.

The chaperone SecB engages nascent secretory proteins and is implicated in SecA-mediated posttranslational targeting^44,45^. Nevertheless, neither disruption of the SecB-binding domain in SecA nor deletion of the *secB* gene substantially affected the substrate pool of SecA (Fig. 5c) or the timing of cotranslational SecA engagement on secretory proteins (Fig. 5a, b and Supplementary Fig. 7a, b), consistent with previous studies that only identified a small number of strictly SecB-dependent preproteins^19,47^. In addition, as we did not observe prolonged SecA binding after deletion of SecB or disruption of the SecA-SecB interaction (Fig. 5a, b and Supplementary Fig. 7a, b), our data do not support a previous model in which SecB releases SecA from the nascent chain^49^. Together, these results indicate that cotranslational SecA engagement on most secretory proteins is largely independent of SecB. On the other hand, SecA binding on long periplasmic loops of translocating IMPs is reduced upon the deletion of SecB or disruption of its binding with SecA, suggesting that SecB could facilitate the formation of high affinity RNC-SecYEG-SecA complexes during cotranslatonal IMP translocation.

TF is an abundant and conserved cotranslational chaperone that forms a molecular cradle for nascent polypeptides at the ribosome exit site and, together with DnaJ/K^61^, ensures the proper folding of cytosolic proteins in bacteria. TF also associates with nascent secretory proteins, and its deletion leads to accelerated preprotein export and increased cotranslational targeting^21,23^. The mechanism behind these observations and the precise role of TF in protein secretion is not well understood. Here we found that TF deletion leads to premature cotranslational associations of SecA with nascent secretory proteins that otherwise bind TF first (Fig. 4j), including those that are devoid of cotranslational SecA binding in wildtype cells (Supplementary Fig. 6e–h). This suggests a competition between TF and SecA in RNC binding in vivo, consistent with their overlapping docking sites on the ribosome^24,25,62^. On the other hand, SecA engagement with the large periplasmic loops of IMPs is compromised in the absence of TF (Supplementary Fig. 6m, n). These observations suggest a role of TF as a master regulator that delays SecA engagement on secretory proteins with weakly hydrophobic signal sequences and thus enforces the posttranslational mode of their targeting. This prioritizes the limited pool of SecA for substrates that require a strictly cotranslational mode of translocation. TF is the only cotranslational protein biogenesis factor whose concentration is stoichiometric with the ribosome^63^ and may therefore be particularly suited to serve this role. In addition, cotranslational protein translocation is limited by translation rate and thought to be much slower than posttranslational translocation^2,53^. Considering the limited number of SecYEG in the cell^63^, TF-induced enforcement of posttranslational translocation may also reduce preprotein residence time on SecYEG, which can be beneficial to cells.

Previous work showed that SRP strongly prefers IMPs with hydrophobic TMDs as the targeting signal as well as secretory proteins with highly hydrophobic signal sequences^4^. Our analyses here further show that secretory proteins are targeted via the SecA-first cotranslational targeting pathway or the TF-first posttranslational pathway based on the hydrophobicity of the signal sequence. These observations support a model in which non-cytosolic substrates are triaged into distinct targeting routes based on the hydrophobicity of the targeting signal. Additional features of the nascent chain, such as helical propensity and potential targeting signals in the mature domain^64^, could also play important roles in substrate triage. Nevertheless, considering that only the signal sequence and the following ~50 amino acids are exposed at the time of TF/SecA engagement, the signal sequence may be one of the most important determinants for nascent protein triage.

On the other hand, overlap in the specificity of SecA with that of SRP and TF enables SecA to provide a backup targeting pathway when the other targeting factor or protein chaperone is deleted. The extensive early association of SecA on TF-first secretory proteins upon TF deletion (Fig. 4j) and the faster export observed in the absence of TF^22^ provides a salient example of this redundancy. Another example is observed with the IMP RodZ. The SeRP profiles (Supplementary Fig. 4c) showed that SRP binds the nascent RodZ TMD as soon as it emerges from the ribosome, followed by SecA binding at the membrane after SRP dissociation, demonstrating SRP-dependent targeting and SecA-dependent translocation of RodZ in WT cells. However, RodZ biogenesis in vivo is weakly dependent on SRP and independent of SRP receptor^32^, and SecA alone is sufficient to reconstitute RodZ insertion into the membrane^31^, suggesting that SecA provides a backup targeting and translocation route for RodZ in the absence of SRP^65^. Taken together, these overlapping specificities enable the distinct targeting pathways to form a robust network that minimizes protein biogenesis defects when one of the pathways is disrupted.

**Fig. 7:**
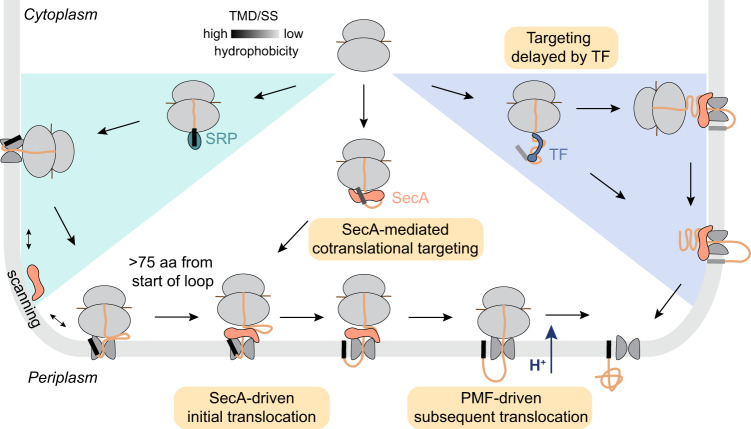
Model of cotranslational SecA engagement during protein transport. Proteins with highly hydrophobic N-terminal signals, including most IMPs and a few secretory proteins, are recognized and targeted to the membrane by SRP. Large periplasmic loops on IMPs cannot be translocated across SecYEG, and thus accumulate at the cytoplasmic surface and disrupts the ribosome-SecYEG junction when >75 amino acids of the periplasmic loop is translated. SecA then gains access to these stalled translocating complexes, uses its ATPase cycle to drive the initial translocation of the loop across SecYEG and restores the ribosome-SecYEG junction. Subsequence translocation is driven by PMF or other forces. Secretory proteins with weakly hydrophobic signal sequences are recognized by TF at a nascent chain length of ~100 amino acids. TF delays their engagement by SecA until protein synthesis is close to complete, thus enforcing a mechanistically posttranslational mode of their export. The other ~25% of secretory proteins with more hydrophobic signal sequences engage with SecA at a nascent chain length of ~100 amino acids. They are possibly targeted to the membrane by SecA, and are translocated across SecYEG in a manner analogous to the translocation of large periplasmic loops on IMPs.

In summary, our work reveals multiple roles of SecA and shows that protein transport pathways in bacteria are organized hierarchically based on the hydrophobicity of the targeting signal (Fig. 7). Proteins with a highly hydrophobic N-terminal targeting signal, which include most IMPs and a few secretory proteins, are recognized and targeted to the plasma membrane by SRP upon emergence of their first TMD or signal sequence (Fig. 7, left). While the cotranslational insertion of TMDs and short periplasmic loops occurs independently of SecA, translation elongation appears insufficient to drive the translocation of large periplasmic domains across SecYEG, leading to the accumulation of these regions at the cytoplasmic surface of the translocating complex and disruption of the ribosome-SecYEG junction. SecA, which rapidly diffuses on the membrane and scans translocating RNCs, recognizes these topologically constrained translocating complexes, likely via a combination of interactions with the exposed periplasmic region, the ribosome, and SecYEG. Using its ATPase cycle, SecA drives the translocation of the periplasmic regions across SecYEG. This restores the ribosome-SecYEG junction and allows continued translocation to be driven by PMF, translation elongation, and other forces. In contrast, secretory proteins that fold slowly in the cytosol harbor weakly hydrophobic signal sequences, which cotranslationally recruit TF after ~100 amino acids are synthesized (Fig. 7, right). TF binding delays the engagement of SecA on these substrates until synthesis of the nascent polypeptide is close to complete, thus enforcing a posttranslational mode of their targeting and translocation to the plasma membrane. The other ~25% of secretory proteins fold rapidly in the cytosol and evolved more hydrophobic signal sequences to cotranslationally engage SecA, and are likely targeted and translocated by SecA in a manner analogous to the translocation of large periplasmic loops of IMPs (Fig. 7, middle). This model highlights how multiple ribosome-associated chaperones and targeting factors together form a robust network of targeting routes that reinforce one another, which allows bacteria to meet the export demands for 1/3 of its proteome that have diverse biophysical properties.

## Methods

### Strain construction

*E. coli* K-12 strain W3110 was used for this study. A thrombin-Avi tag (GLVPRGSGSGSGLNDIFEAQKIEWHE) was chromosomally fused to the C-terminus of SecA using CRISPR^66^. For gene deletions, a cassette containing kanamycin resistance marker was amplified from pKD4 plasmid and inserted into the target locus to replace the WT SecA gene^67^. For construction of a strain harboring OppC-43aa repeat, a silently mutated DNA fragment (AGTCAATTCGCGTATGATGACACGGATTGGGCCATGATGAGCTCTGCGCCAGACATGGAAAGCGGCCATTATTTCGGCACCGATAGCAGCGGCCGTGATTTGCTGGTCCGTGTGGCCATCGGTGGCCGA) encoding the first periplasmic loop of OppC (bp 178-306) was synthesized and chromosomally inserted into OppC using CRISPR^66^. For construction of AcrB-∆71aa, WT AcrB was cloned into a plasmid and AcrB 50-120 amino acids was deleted by amplifying the plasmid using oligonucleotides 5’ GATCTCCGCCTCCTACGAAGTTCAGCAGCAAGGGGT3’ and 5’ CTTGCTGCTGAACTTCGTAGGAGGCGGAGATCGTTA3’. Genomic AcrB was then replaced with AcrB-∆71aa using CRISPR^66^. All strains were confirmed by amplification of genomic DNA and sequencing.

### Purification of SecA-RNCs for SeRP

400 mL of *E.* coli cells were grown in LB medium at 37 °C to an OD600 of 0.4 and harvested by rapid filtration through nitrocellulose membranes of 0.2 mm in pore size, followed by flash freezing in liquid nitrogen. For CCCP-treated cells, CCCP (Sigma) was added into the cell culture to a final concentration of 50 µM immediately before filtering such that the treatment time is approximately 2.5 min. Frozen cells were mixed with 1 mL frozen Lysis Buffer (50 mM HEPES pH 7.0, 100 mM NaCl, 10 mM MgCl_2_, 5 mM CaCl_2_, 1 mM chloramphenicol, 1 mM PMSF, 50 U/ml DNaseI (recombinant DNaseI, Roche)) and lysed by mixer milling (2 min, 25 Hz, Retsch). To increase the yield of purified SecA-RNCs, 1.6 L of cells were cultured and lysed in four batches. Frozen lysed cell powder was mixed, and half of the powder was added in batches to 4 mL of Lysis Buffer EDC (50 mM HEPES pH 4.5, 100 mM NaCl, 10 mM MgCl_2_, 5 mM CaCl_2_, 1 mM chloramphenicol, 1 mM PMSF) supplemented with 20 mM EDC with constant stirring at room temperature. Once the cell powder was completely dissolved, EDC was added again. The other half of the frozen cell powder was then added as described above. The final concentration of EDC is 20 mM. The thawed lysate was stirred for an additional 5 min after the powder was completely dissolved. The crosslinking reaction was quenched with 20 mM glycine, 100 mM Tris pH 8.0, 4 mM NaHCO_3_ and incubated for 5 min at room temperature with constant stirring. After quenching, the lysate was further incubated with 1% Triton X-100 for 5 min and the concentration of MgCl_2_ and CaCl_2_ was adjusted to 10 mM and 5 mM, respectively, to account for the volume increase. The RNA concentration was determined, and polysomes supplemented with 100 U/mL SUPERase*In (Ambion) were digested using MNase (3750 U/1 mg RNA) for 15 min at room temperature. The reaction was terminated by addition of 6 mM EGTA and chilling on ice. Unbroken cells were removed by centrifugation at 3000 rpm for 6 min at 4 °C. Monosomes were purified by centrifugation through sucrose cushion (30% sucrose, 50 mM Tris pH 7.4, 150 mM NaCl, 10 mM MgCl_2_, 1 mM chloramphenicol, 1x protease inhibitors (Complete EDTA-free, Roche), 0.4% Triton X-100, 0.1% NP-40) in a TLA100.3 rotor at 80,000 rpm for 100 min at 4 °C. Pellets were washed once and resuspended in Wash Buffer (50 mM Tris pH 7.4, 150 mM NaCl, 10 mM MgCl_2_, 1 mM chloramphenicol, 1 mM PMSF, 0.4% Triton X-100, 0.1% NP-40).

20 µg of total RNA were removed from resuspended monosomes for ribosome profiling of the total translatome. To the rest, 100 µL Pierce Streptavidin Magnetic Beads (pre-washed for 3 times in Wash Buffer) was added per 3 mg RNA, and the suspension was rotated for 1 h at 4 °C. Bead were washed 2 × 10 min at 4 °C and 3 × 5 min at room temperature in Wash Buffer. After the fifth wash, beads were re-equilibrated in Cleavage Buffer (50 mM Tris pH 7.4, 150 mM NaCl, 10 mM MgCl_2_, 1 mM chloramphenicol, 0.01% Triton X-100). Thrombin cleavage was performed by mixing the beads with 400 µL of Cleavage Buffer plus 8 U of thrombin (Cytiva) and incubating on a nutator for 30 min at room temperature. The eluate was used for subsequent RNA extraction.

### Library preparation

Libraries are prepared as described in^68^ with modifications. All steps were performed in non-stick, RNase free microfuge tubes (Ambion). RNA from total monosomes and SecA-bound monosomes was extracted using Direct-zol kit (Zymo) according to the manufacturer’s instructions. 3 µg of RNA from each sample was mixed with 2x Novex TBE-Urea sample buffer (Invitrogen) and denatured for 2 min at 80 °C. Samples were loaded onto a home-made 15% polyacrylamide TBE-Urea gel and run for 50 min at 200 V in 1x TBE. The gel was stained for 5 min with SYBR-gold (Invitrogen) and the ribosome footprints were isolated by excising the 15 nt - 45 nt region as indicated by the 10 bp ladder (Invotrogen) and a 45 nt oligo from the gel. The gel pieces were placed into 0.5 mL gel breaker tubes (IST Engineering), nested into a 1.5 mL tube and centrifuged for 5 min at 20,000 x g. 500 µL of RNA extraction buffer (300 mM NaOAc pH 5.5, 1 mM EDTA pH 8.0) supplemented with 100 U/mL SUPERase*In was added to each sample, and the tubes were shaken overnight in a thermomixer at 1400 rpm, 4 °C. Gel pieces were transferred to a Spin-X cellulose acetate column (Fisher) and centrifuged at 20,000 x g for 5 min. The flow through was mixed with 1.5 µL Glycoblue and 600 µL isopropanol and incubated for 1 h on dry ice to precipitate RNA. The samples were centrifuged for 30 min at 20,000 x g, 4 °C and the pellets were washed with ice-cold 80% ethanal followed by resuspended in 4 µL of 10 mM Tris pH 8.0. For dephosphorylation, 3.5 µL of sample was denatured for 2 min at 80 °C followed by quick cooling on ice, mixed with 0.5 µL 10x T4 polynucleotide kinase buffer (NEB), 0.5 µL T4 polynucleotide kinase (NEB) and 0.5 µL SUPERase*In, and incubated for 1 hr at 37 °C. For linker ligation, 3.5 µL 50% w/v PEG-8000, 0.5 µL 10x T4 RNA ligase 2 buffer (NEB), 0.5 µL 20 μM Universal miRNA cloning linker (NEB) and 0.5 µL T4 RNA ligase 2 truncated (NEB) were added into each sample and incubated for 3 h at 30 °C. To deplete unligated linker, 0.5 µL Yeast 5’-deadenylase (NEB) and 0.75 µL RecJ exonuclease (Epicentre) were added after linker ligation and the reaction was further incubated for 45 min at 30 °C. The ligation products was purified with Oligo and Concentration Kit (Zymo) and eluted in 10 μl of nuclease-free H_2_O. For reverse transcription, the sample was mixed with 2 µL 1.25 µM RT primer and denatured at 65 °C for 5 min, then mixed with 4 µL 5x RT buffer (Invitrogen), 1 µL10 mM dNTPs, 1 µL 0.1 M DTT, 1 µL SUPERase*In and 1 µL Superscript III RT (Invitrogen). Samples were incubated for 30 min at 50 °C followed by addition of 2.3 µL of 1 M NaOH and incubation for 20 min at 70 °C to hydrolyze RNA. Samples were loaded onto a home-made 10% polyacrylamide TBE-Urea gel and run for 60 min at 200 V in 1x TBE. The gel was stained for 5 min with SYBR-gold, the bands between 111 nt – 141 nt were excised, and nucleic acids were extracted as described earlier, except that DNA extraction buffer was used (300 mM NaCl, 1 mM EDTA pH 8.0, 10 mM Tris pH 8), and the DNA was resuspended in 15 µL of 10 mM Tris pH 8.0. For circularization, 2 µL 10x CircLigase buffer (Epicentre), 1 µL 1 mM ATP, 1 µL 50 mM MnCl_2_ and 1 µL CircLigase (Epicentre) were added and incubated for 1 h at 60 °C. To inactivate the enzyme, the reaction was further incubated for 10 min at 80 °C. For PCR amplification, 4 µL of circularized DNA was mixed with 4 µL 10 µM barcoded primer, 49.4 µL nuclease-free water, 16 µL 5x HF buffer (NEB), 1.6 µL of 10 mM dNTPs, 4 µL forward primer and 1 µL Phusion polymerase (NEB), and the PCR reaction was run for 6–10 cycles. Samples were loaded onto a home-made 8% polyacrylamide native TBE gel and run for 1 hr at 120 V in 1x TBE. The gel was stained for 5 min with SYBR-gold, and the bands at ~170 bp were excised. The DNA product are extracted as described earlier followed by resuspension in 10 µL of 10 mM Tris pH 8. The libraries were quantified by Qubit and sequenced on a Hiseq 2500.

### Fractionation-coupled ribosome profiling

400 mL of *E. coli* cells were harvested and lysed as described earlier. Frozen cell powder was thawed in 1 mL of Lysis Buffer (50 mM HEPES pH 7.0, 100 mM NaCl, 10 mM MgCl_2_, 5 mM CaCl_2_, 1 mM chloramphenicol, 1 mM PMSF) at room temperature and immediately centrifuged at 3000 rpm for 6 min at 4 °C to remove unbroken cells. After quantifying RNA concentration, MNase digestion was performed as described earlier followed by centrifugation in a TLA120.2 rotor at 30,000 rpm for 20 min at 4 °C. The supernatant was saved as cytosolic fraction. The pellet was washed and resuspended with a dounce homogenizer in Resuspension Buffer (50 mM HEPES pH 7.0, 100 mM NaCl, 10 mM MgCl_2_, 5 mM CaCl_2_, 1 mM chloramphenicol, 1 mM PMSF, 1% Triton X-100, 6 mM EGTA). Insoluble material was removed by centrifugation in a TLA120.2 rotor at 30,000 rpm for 20 min at 4 °C, and the supernatant was saved as the membrane faction. Monosomes from both the cytosolic and membrane factions were purified, RNA was extracted, and sequence libraries were generated and sequenced as described earlier.

### Western blotting

For affinity purification analysis, total monosomes were collected, and affinity purification was performed as described earlier except that the cleavage step was omitted. To concentrate the SecA-RNCs, the beads were mixed with a small volume of 5x SDS sample buffer after washing and boiled for 5 min to elute SecA-RNCs. Total monosomes were mixed with 5x SDS sample buffer and boiled for 5 min. Samples were resolved on 12.5% or 15% Tris-glycine gels, transferred to nitrocellulose membrane (Bio-Rad), and probed with rabbit anti-SecA antibody (a gift from Tom A. Rapoport, 1:1000 dilution) and mouse anti-S13 antibody (DSHB, 1:3000 dilution). Primary antibodies were incubated with IRDye 800CW Goat anti-Mouse and Goat anti-Rabbit secondary antibodies (LI-COR, 1:15000 dilution) for detection. The membranes were scanned by Odyssey Imager (LI-COR) and the images were processed using ImageJ.

For cell fractionation analysis, cell fractionation was performed as described earlier. Total lysate, supernatant and resuspended pellet were collected and resolved on 12.5% or 15% Tris-glycine gels. YidC and DnaK were detected by western blot using rabbit anti-YidC antibody (a gift from Ross E. Dalbey, 1:5000 dilution) and mouse anti-DnaK antibody (Abcam, 1:3000 dilution). Purified 70 S ribosomes were subjected to centrifugation at different speeds indicated in Fig. S2a. Ribosomal proteins were detected by silver staining.

### Purification of MNase

MNase purification was performed following the protocol described in^69^. Briefly, *E. coli* cells overexpressing mature His_6_-tagged MNase were lysed in lysis buffer (50 mM Tris pH 7.5, 25 mM NaCl, 1 mM CaCl2, 5 mM imidazole pH 8.0, 1 mM PMSF, 5% glycerol, 1x Halt protease inhibitor) by French press followed by centrifugation at 31,000 x g for 30 min in JA20 rotor. Clarified lysates were loaded onto Ni-sepharose resin column equilibrated in Ni buffer (50 mM Tris pH 7.5, 25 mM NaCl, 1 mM CaCl_2_, 5 mM imidazole pH 8.0, 5% glycerol). The resin was extensively washed using washing buffer (50 mM Tris pH 7.5, 250 mM NaCl, 10 mM imidazole pH 8.0, 5% glycerol) with a final wash in Ni buffer to adjust salt concentration. The protein was eluted using elution buffer (50 mM Tris pH 7.5, 25 mM NaCl, 250 mM imidazole pH 8.0, 5% glycerol) and dialyzed overnight against dialysis buffer (50 mM Tris pH 7.5, 25 mM NaCl, 1 mM EDTA, 5% glycerol). His6-tagged MNase was concentrated to ~14 mg/mL and the MNase activity was determined by performing the MNase activity assay as previously described^69^. Aliquots were stored at −80 °C.

### Data analysis

Adaptor sequences were trimmed from sequencing reads using Cutadapt. Reads were mapped to bacterial genome using Bowtie after discarding the reads mapping to ribosomal RNAs (Supplementary Table 1). The *E.coli* W3110 reference genome assembly (ASM1024v1) was downloaded from EnsemblBacteria (https://bacteria.ensembl.org). Ribosome density was assigned to 14-nt upstream of the 3’-end of reads using reads with size range 15–45 nt as described elsewhere^70^ to reach single-codon resolution. Nucleotide reads at each codon were then summed and used for all additional analyses.

#### Gene-level enrichment

For each gene, the sum of raw reads and RPM-normalized reads at each codon, excluding the first five and last five codons, were calculated for both translatome and SecA interactome. Only the genes with greater than 100 reads in both biological replicates of translatome and SecA interactome were included. SecA enrichment on each gene was calculated as the ratio of RPM-normalized reads from SecA interactome to that from translatome.

#### Positional enrichment

For each gene, the enrichment at each codon was calculated as the ratio of the normalized reads over a window of 7 residues in the SecA interactome over that in the translatome. For the heatmaps in Figs. 2d and 4a, log2 enrichment was calculated at each codon. The localization score at each codon was calculated as the ratio over a window of 11 residues of reads in membrane fraction and that in soluble fraction to normalize for local variations in translation speed.

For metagene analyses of SecA enrichment, the first five and last five codons were excluded and only the genes that had an average reads per codon >0.5 in both translatome and SecA interactome were used. Reads at each codon are first smoothed using a 5 residue rolling average and genes were normalized to their expression level by dividing the reads at each codon by the average reads per codon of the respective gene. ORFs from specific subsets (e.g. Sec substrates) are then aligned to the start codon or to the N-terminus of initial TMD/signal sequence as indicated and the mean of normalized reads and bootstrapped 95% CI were calculated at each position. For metagene analyses of ribosome membrane association, localization score at each codon is divided by the mean localization score of the respective gene. ORFs are aligned as indicated and the median of normalized localization scores and bootstrapped 95% CI were calculated at each position.

#### Loop analysis

The length of each periplasmic loop and cytoplasmic loop was calculated based on the predicted topology of each inner membrane protein. Periplasmic or cytoplasmic loops longer than 100 amino acids are aligned to their N-terminus, except for the loops N-terminal to the first TMD, which usually are not targeted to the membrane during their synthesis. After excluding the loops from the genes with a low read coverage (average reads per codon <0.5), we continued with 122 large periplasmic loops and 95 large cytoplasmic loops. For both replicates of translatome and SecA interactome, reads at each codon on the loops are smoothed using a 5 residue rolling average and divided by the average reads per codon of the gene harboring the loop, to normalize for the expression level of the gene. The mean of normalized reads and bootstrapped 95% CI were calculated at each position. When comparing the SecA enrichment after the emergence of a large periplasmic loop between CCCP-treated and untreated cells (Fig. 6a), we included the residues downstream of the periplasmic loops in the metagene analyses of both cells because of the observed prolonged SecA binding after CCCP treatment, by aligning the genes to the N-terminus of its predicted periplasmic loops and calculating the mean of normalized reads and bootstrapped 95% CI at each position.

#### Peak detection

To identify SecA binding peaks, we developed an algorithm to scan for the stretches of residues with a high SecA enrichment. We first excluded the genes with a low read coverage or a low correlation between replicates by applying three thresholds. Only the genes that passed the following thresholds after excluding the first five and last five codons were considered for further peak detection analysis. First, the sum of reads in both replicates of translatome and SecA interactome >100. Second, the average reads per codon >0.5 in both replicates of translatome and SecA interactome. Third, the Pearsons’s correlation coefficient (Pearson’s r) between replicates >0.5 in both translatome and SecA interactome.

We proceeded to peak detection analysis with genes fulfilling all these requirements. The ratio over a window of 5 residues of normalized reads in SecA interactome and translatome for both replicates was calculated. We defined the SecA binding peaks as the regions that met the following criteria: (1) SecA enrichment > = 1.7-fold for at least 12 consecutive codons in both replicates. (2) The overlap of the peaks from two replicates > = 6 codons. (3) The position of the peak >30 codons to avoid the detection of anomalous peaks caused by the known ribosome profiling technicalities and because we found SecA binding requires the emergence of the nascent chain. When identifying the strong SecA binding peaks (Figs. 3g and S7a), we increased the threshold of SecA enrichment from 1.7-fold to 3.5-fold to avoid the detection of SecA binding peaks induced by SecA scanning on the membrane.

#### WebLogo analysis

WebLogo analyses to compare the amino acid compositions of signal sequences of SecA-first substrates, TF-first substrates and non-SecA substrates were performed using the WebLogo3 (http://weblogo.threeplusone.com)^38^, with the signal sequences aligned to the second amino acid from their N-terminus.

#### Gene categorization and protein topology predictions

Genes were categorized based on their GO (gene ontology) annotations taken from Ecocyc^71^. Secretory proteins are composed of periplasmic proteins, outer membrane proteins, lipoproteins and extracellular proteins. The topology of inner membrane proteins was predicted by TOPCONS^72^ and TMHMM^73^ unless it has been experimentally verified and deposited in Uniprot^74^. The signal sequences of secretory proteins were predicted by SignalP^75^.

#### Hydrophobicity determination

The hydrophobicity of signal sequences was assessed by calculating the average Kyte-Doolittle hydrophobicity with a 10 amino acids rolling window. The Kyte-Doolittle value of the most hydrophobic window was recorded as the hydrophobicity of the signal sequence.

#### Contact order calculation

The predicted structures of SecA-first and TF-first substrates were downloaded from AlphaFold Protein Structure Database (https://alphafold.ebi.ac.uk/)^76,77^. The absolute contact order for each protein is calculated based on the structure, as previously described^43^.

### Quantification and statistical analysis

All analysis was performed in python. Statistical significance in comparing the distributions of SecA enrichment (Fig. 3e) and hydrophobicity (Fig. 4k and Supplementary Fig. 6j) was determined using Wilcoxon rank-sum tests. None of the experiments involved blinding or randomization. The number of independent biological replicates used for an experiment and *p*-vales are indicated in the figure legends.

### Reporting summary

Further information on research design is available in the Nature Research Reporting Summary linked to this article.

## Supplementary information

Supplementary information

Description of Additional Supplementary Files

Supplementary Data 1

Reporting Summary

## Data Availability

The data supporting the findings of this study are available from the corresponding authors upon reasonable request. The accession number for the data reported in this paper is GSE185572. The protein structures used to calculate absolute contact order were downloaded from AlphaFold Protein Structure Database (https://alphafold.ebi.ac.uk/). Source data for the figures and supplementary figures are provided as a Source Data file. Source data are provided with this paper.

## Source data

Source Data
