# Supplementary material for: Ribosome profiling reveals multiple roles of SecA in cotranslational protein export
Source: Nat Commun. 2022 Jun 13;13:3393. doi: 10.1038/s41467-022-31061-5 (PMC9192764; doi:10.1038/s41467-022-31061-5)
Supplement: Supplementary file 1 — Supplementary information [file 41467_2022_31061_MOESM1_ESM.pdf]

Supplemental Information for

Ribosome profiling reveals multiple roles of SecA in cotranslational protein export

Zikun Zhu,[1,2], Shuai Wang,[1,2,3] and Shu-ou Shan[1,*]

[1]Division of Chemistry and Chemical Engineering, California Institute of Technology, Pasadena, CA 91125, USA

[2]These authors contributed equally

[3]Current address: Department of Molecular and Cellular Physiology, Stanford University, Stanford, CA 94305, USA

*e-mail: sshan@caltech.edu

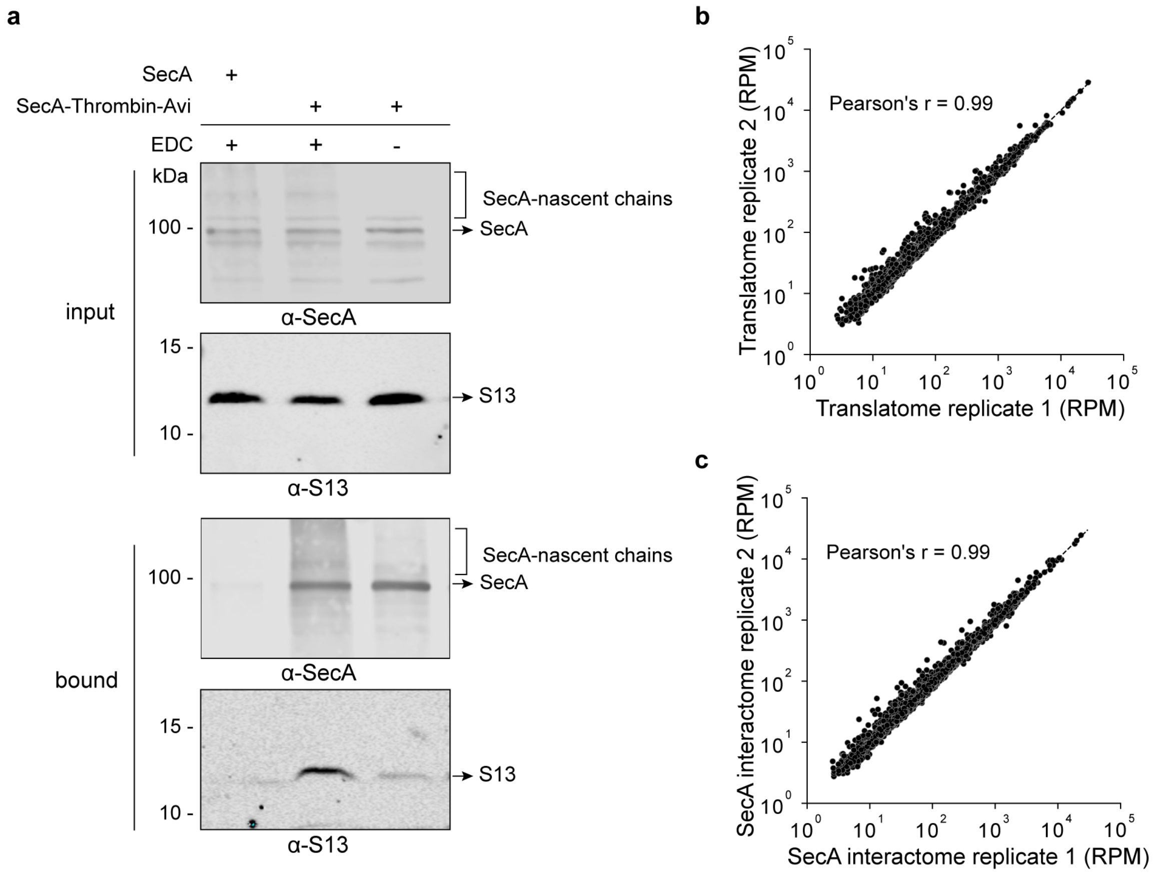

Supplementary Fig. 1 Selective ribosome profiling of SecA.

a, Affinity purification of SecA-RNC complexes analyzed by western blot using antibodies against SecA and the ribosomal protein S13. Representative results from two independent experiments are shown. Source data are provided in the Source Data file. **b,c,** Reproducibility of the translatome and SecA interactome data sets from two biological replicates.

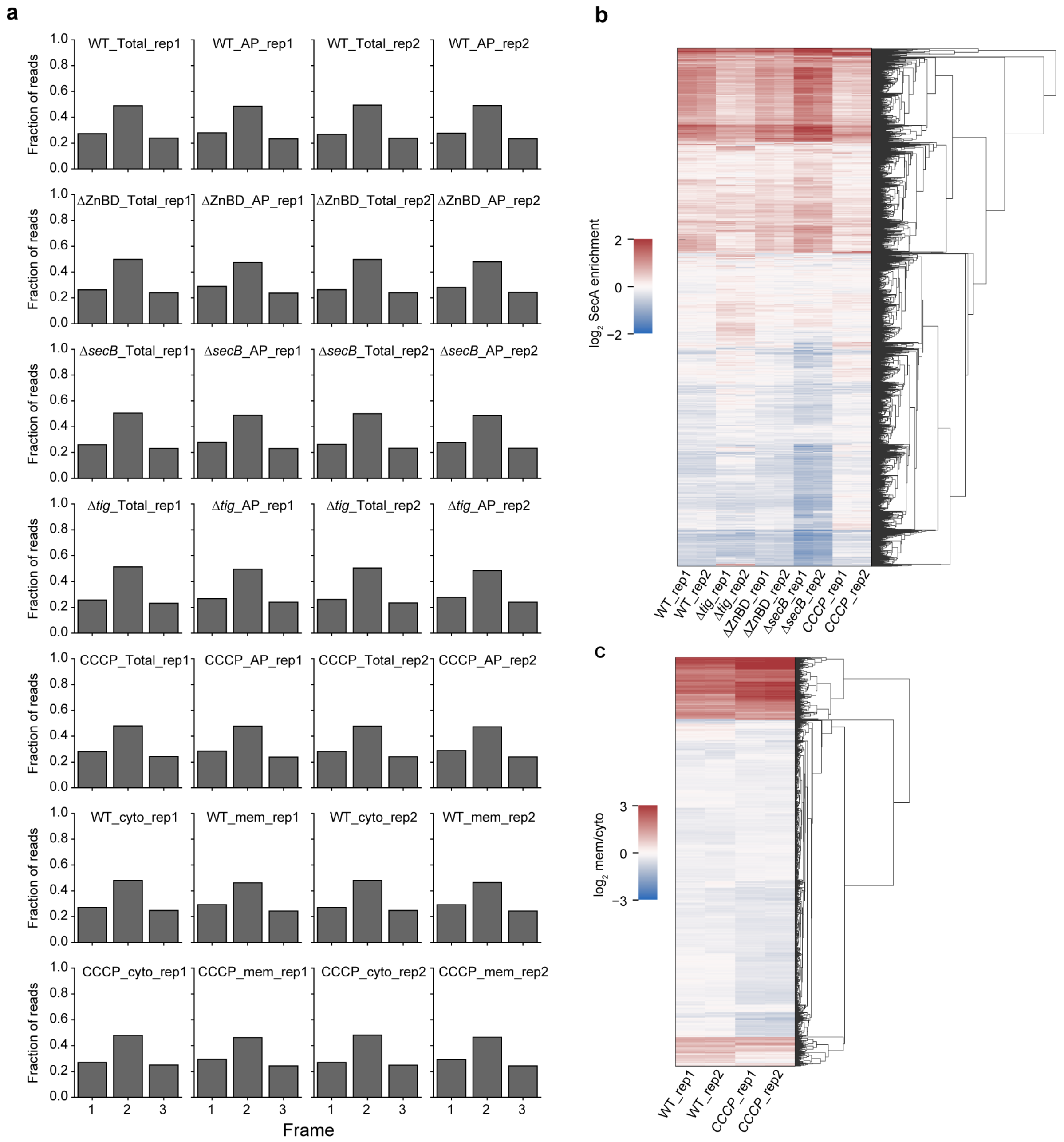

Supplementary Fig. 2 Comparison of all datasets generated in this study.

a, The fraction of reads with P-site at three possible frames. **b,** Hierarchical clustering of gene-level enrichment for all selective ribosome profiling experiments. **c,** Hierarchical clustering of gene-level enrichment for all fractionation-coupled ribosome profiling experiments.

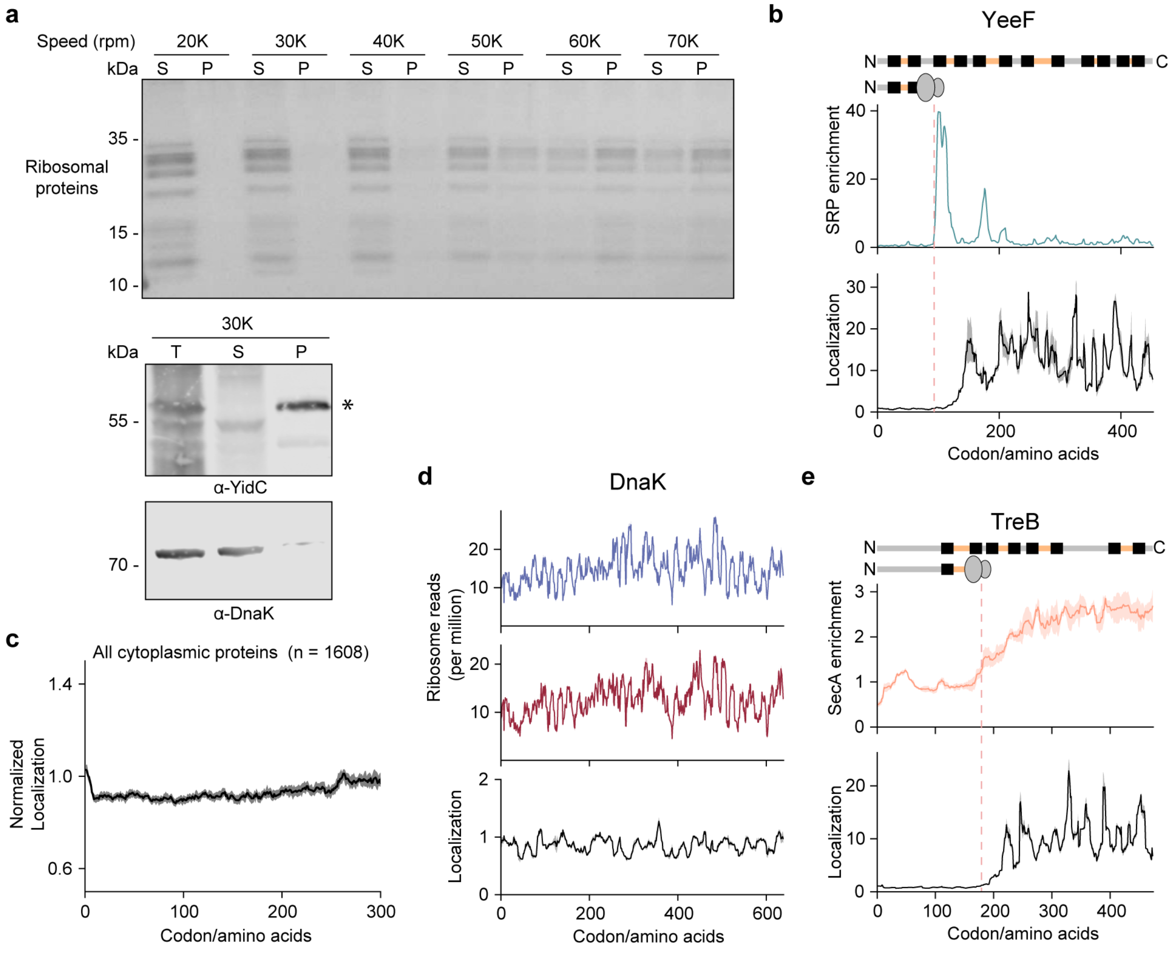

Supplementary Fig. 3 Fractionation-coupled ribosome profiling.

a, Upper panel, centrifugation of purified 70S ribosomes using TLA120.2 rotor at different speeds analyzed by silver stain. The purified 70S ribosomes pelleted at centrifugation speeds above 40K rpm. To avoid the pelleting of ribosomes in cytosolic fraction during cell fractionation, we used 30K rpm, 20 min. S, supernatant; P, pellet. Lower panel, the centrifugation condition we used in fractionation experiment is sufficient to separate cytosolic and membrane fractions as analyzed by western blot using antibodies against YidC (an IMP) and DnaK (a cytosolic protein). The asterisk denotes the YidC protein. T, total lysate; S, supernatant; P, pellet. Representative results from two independent experiments are shown. Source data are provided in the Source Data file. **b,** The SRP interaction profile and ribosome localization profile of YeeF, an IMP whose first TMD was skipped by SRP[4]. Protein topology is shown above and colored as in Fig. 2b. Solid lines show the mean values and shaded areas show the differences between two independent biological replicates. **c,** Metagene ribosome localization profile of all

cytoplasmic proteins aligned to the start codon. Solid lines show the mean values and shaded areas show the 95% confidence interval (CI). **d,** Representative ribosome localization profile of a cytosolic protein. Solid lines show the mean values and shaded areas show the differences between two independent biological replicates. **e,** Representative SecA interaction profile and ribosome localization profile of an IMP. Protein topology is shown above and colored as in Fig. 2b. Solid lines show the mean values and shaded areas show the differences between two independent biological replicates.

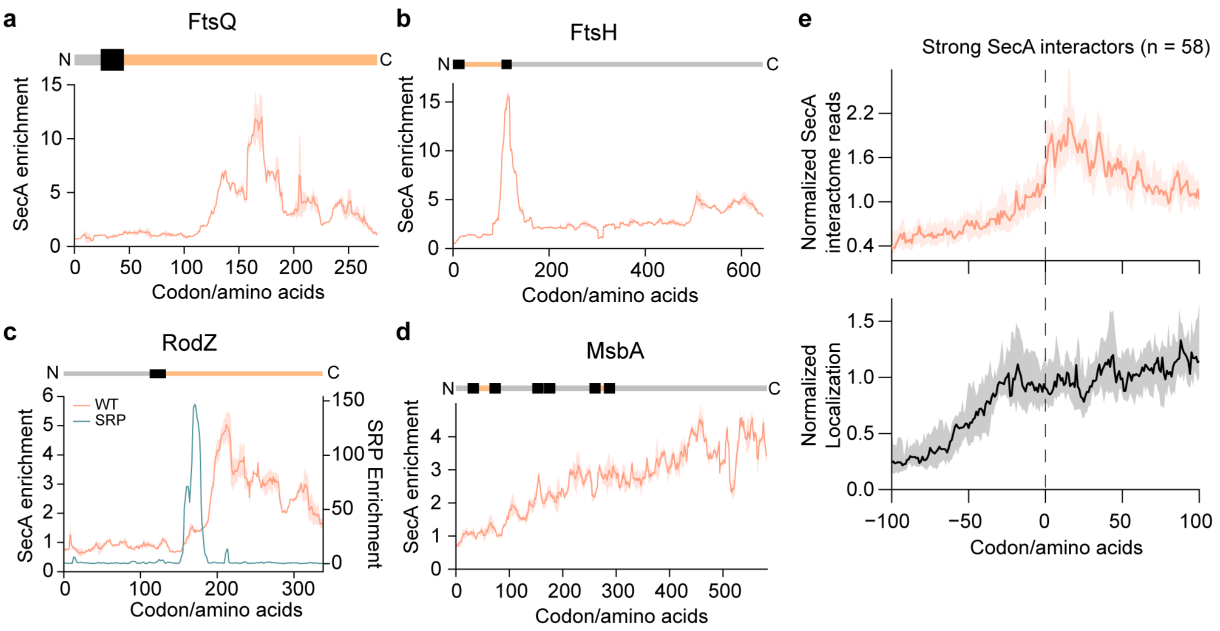

Supplementary Fig. 4 SecA interacts with IMPs containing large periplasmic loops.
a-c, Representative SecA interaction profiles of IMPs with large periplasmic loops. Protein topology is shown above and colored as in Fig. 2b. Solid lines show the mean values and shaded areas show the differences between two independent biological replicates. **d,** SecA interaction profile of a detected SecA strong interactor lacking a periplasmic loop. Its detection is likely due to a higher baseline of SecA enrichment caused by the scanning mode. Protein topology is shown above and colored as in Fig. 2b. Solid lines show the mean values and shaded areas show the differences between two independent biological replicates. **e,** Metagene SecA interactome profile and ribosome localization profile of 58 SecA strong interactors aligned to the onset of SecA binding peaks (position 0). Solid lines show the mean values and shaded areas show the 95% CI.

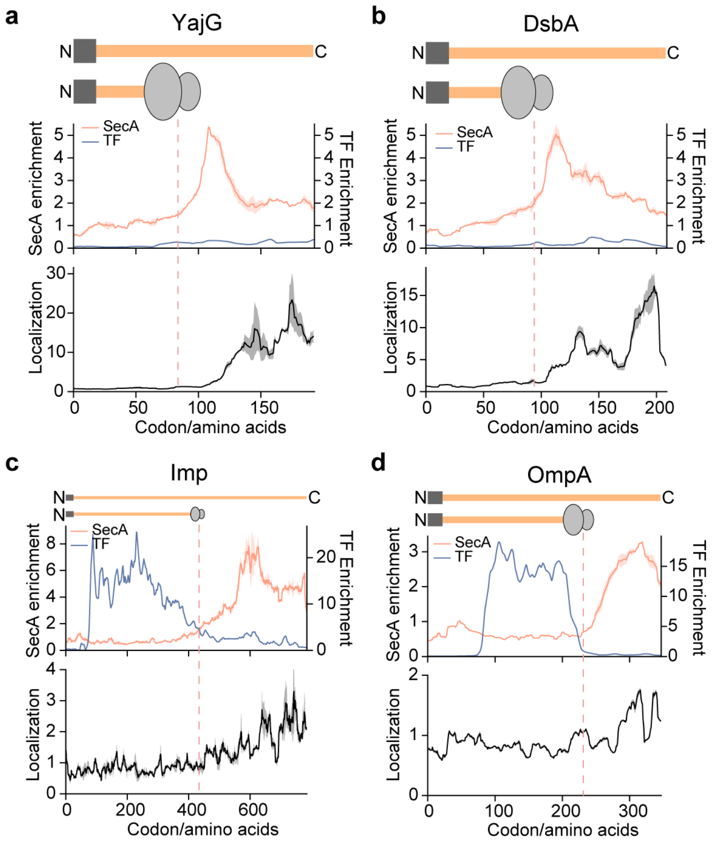

Supplementary Fig. 5 Temporal separation of SecA and TF binding on ribosomes translating secretory proteins.

a,b, Representative SecA and TF interaction profiles, and ribosome localization profiles of SecA-first substrates. Protein topology is shown above and colored as in Fig. 4c. Solid lines show the mean values and shaded areas show the differences between two independent biological replicates. **c,d,** Representative SecA and TF interaction profiles, and ribosome localization profiles of TF-first substrates. Protein topology is shown above and colored as in Fig. 4c. Solid lines show the mean values and shaded areas show the differences between two independent biological replicates.

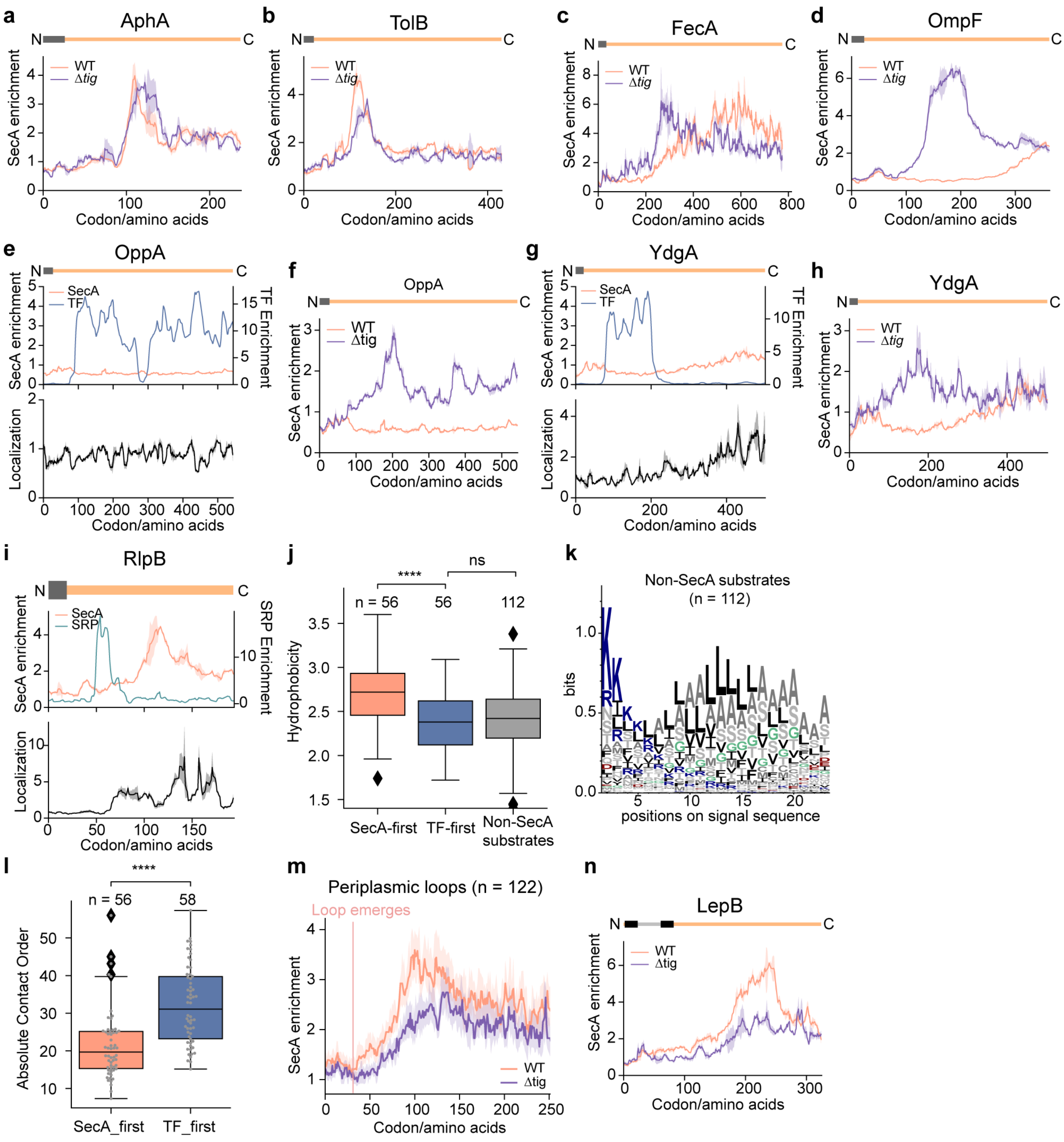

Supplementary Fig. 6 TF deletion leads to earlier SecA engagement on nascent secretory proteins and decreased SecA binding on IMPs.

a-h, Representative SecA interaction profiles of SecA-first substrates (**a,b**), TF-first SecA substrates (**c,d**), and non-SecA substrates (**e-h**). Protein topology is shown above and colored as in Fig. 4c. Solid lines show the mean values and shaded areas show the differences between two independent biological replicates. **i,** Representative SecA and SRP interaction profile, and ribosome localization profile of a SecA-first substrate that recruit SRP cotranslationally[4]. Protein topology is shown above and colored as in Fig. 4c. Solid lines show the mean values and

shaded areas show the differences between two independent biological replicates.

j, Hydrophobicity of signal sequences of SecA-first, TF-first, and non-SecA substrates. The centre line represents the median, the bounds of box represent the upper and lower quartiles, and the whiskers indicate 1.5x the interquartile range. SecA-first vs TF-first: $P = 3.716e^{-6}$; TF-first vs non-SecA substrates: $P = 4.078e^{-1}$, Wilcoxon rank-sum test **k,** WebLogo representations of the amino acid compositions of signal sequences of non-SecA substrates aligned to the second amino acid. **l,** Absolute contact order calculated for SecA-first and TF-first substrates based on the Alphafold-predicted structures of each protein. The structure of FdoG is not deposited in the database and is excluded from the analysis. The centre line represents the median, the bounds of box represent the upper and lower quartiles, and the whiskers indicate 1.5x the interquartile range. $P = 5.042e^{-8}$, two-sided Wilcoxon rank-sum test. Source data are provided in the Source Data file. **m,** Metagene SecA enrichment of large periplasmic loops aligned to their N-terminus. The median values of SecA enrichment in WT strain and in Δ*tig* strain are compared. Shaded areas show the 95% CI. **n,** Representative SecA interaction profile of an IMP with a large periplasmic loop on which SecA binding is compromised upon TF deletion. Protein topology is shown above and colored as in Fig. 2b. Solid lines show the mean values and shaded areas show the differences between two independent biological replicates.

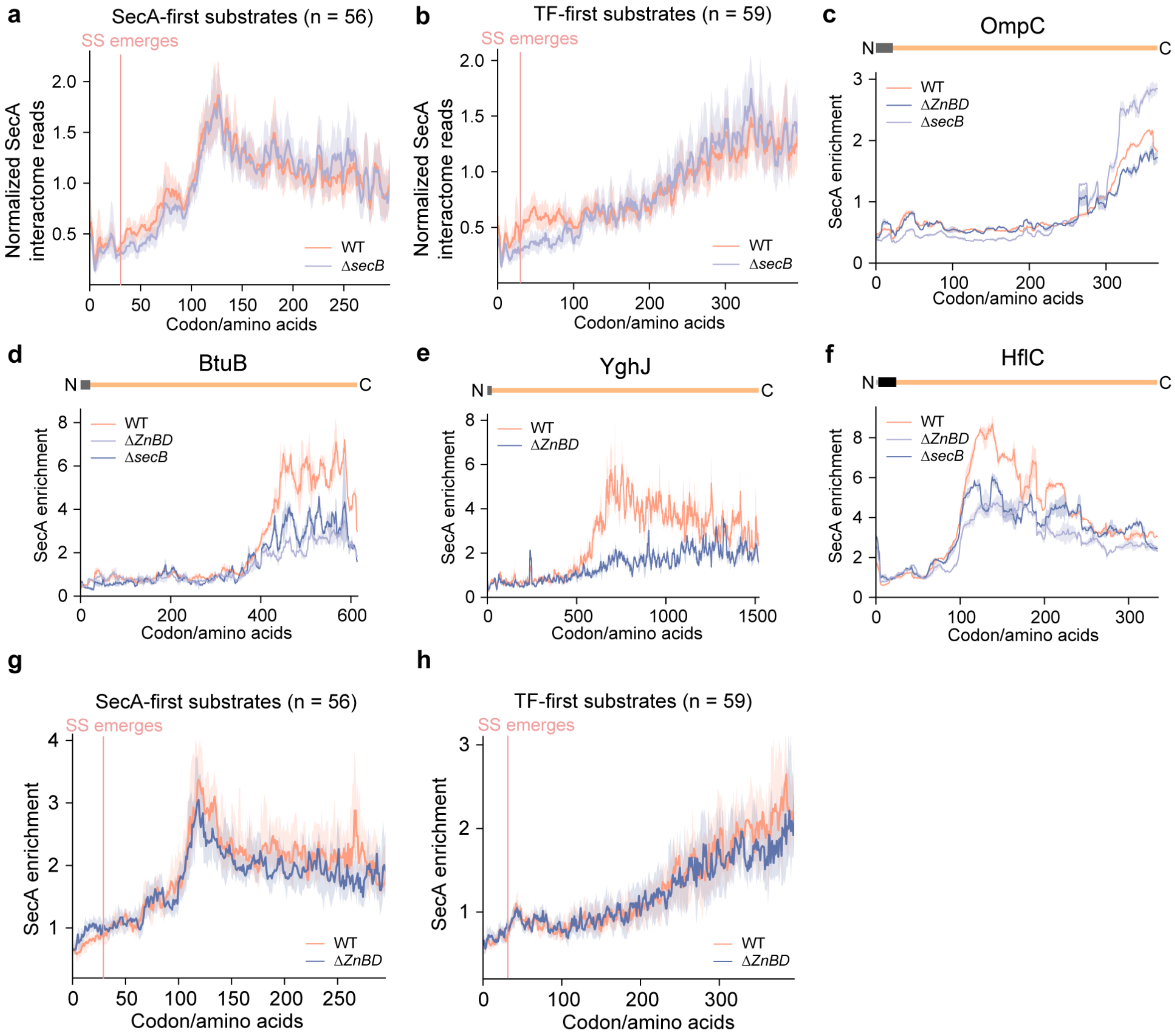

Supplementary Fig. 7 Role of SecB in cotranslational SecA interactions.

a,b, Metagene SecA interactome profile of SecA-first (**a**) and TF-first (**b**) substrates aligned to the N-terminus of signal sequences. Solid lines show the mean values and shaded areas show the 95% CI. **c,** Representative SecA interaction profile of a secretory protein on which the SecA binding peak showed modest changes upon SecB deletion. Protein topology is shown above and colored as in Fig. 4c. Solid lines show the mean values and shaded areas show the differences between two independent biological replicates. **d,e,** Representative SecA interaction profiles of secretory proteins on which SecA binding peaks are severely compromised by the SecB deletion or the SecAΔZnBD mutation. The SecA interaction profile for YghJ from the Δ*secB* strain is noisy and therefore not shown in (**e**). Protein topology is shown above and colored as in Fig. 4c. Solid lines show the mean values and shaded areas show the differences between two independent biological replicates. **f,** Representative SecA interaction profile of an

IMP on which SecA binding is compromised. Protein topology is shown above and colored as in Fig. 2b. Solid lines show the mean values and shaded areas show the differences between two independent biological replicates. **g,h,** Metagene SecA enrichment of SecA-first (**g**) and TF-first (**h**) substrates aligned to the N-terminus of signal sequences. The median values of SecA enrichment in WT strain and in Δ*ZnBD* strain are compared. Shaded areas show the 95% CI.

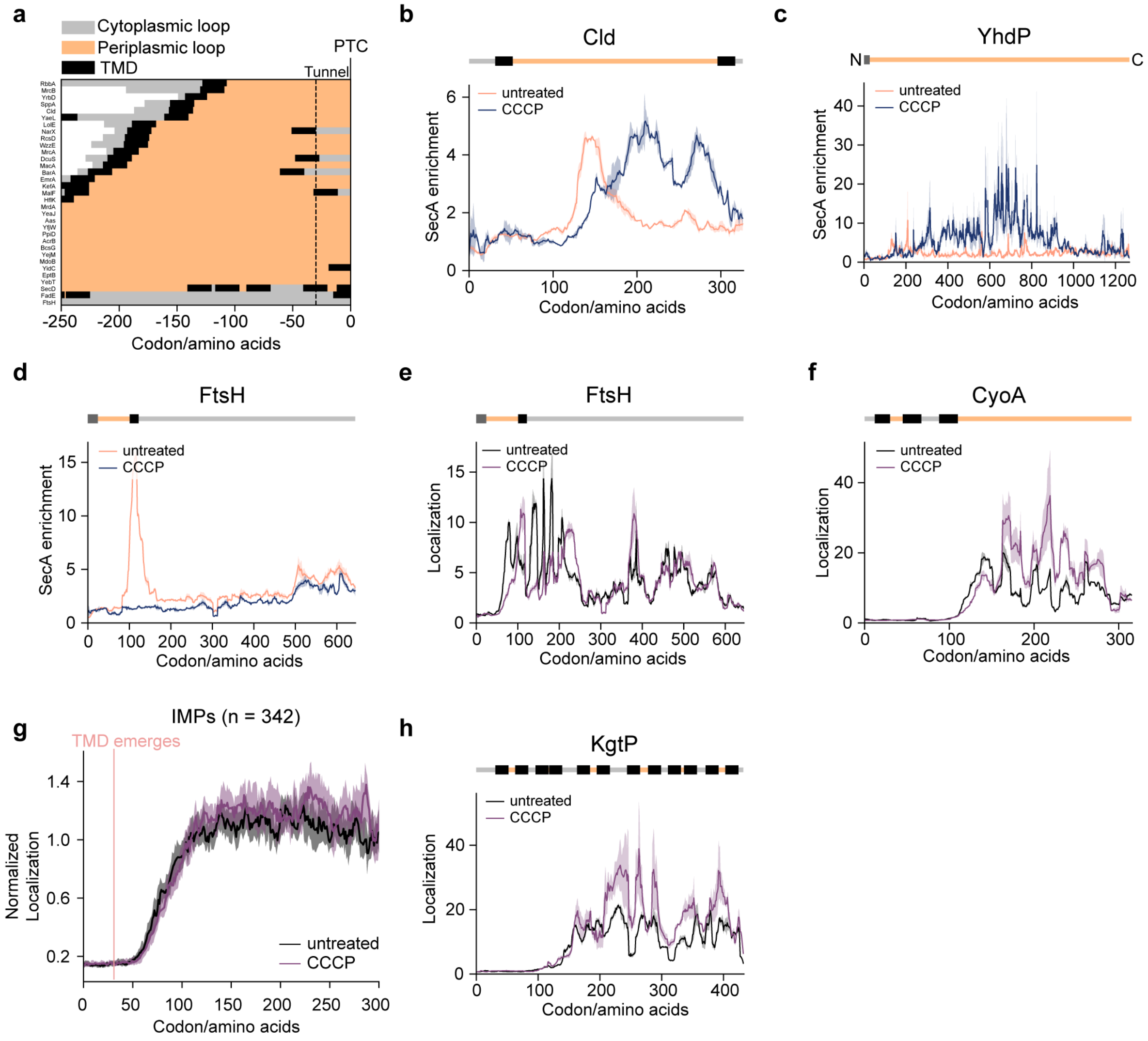

Supplementary Fig. 8 Loss of PMF leads to desregulated SecA binding.

a, Topology of translated nascent chains of strong SecA interactors, aligned to their C-terminus (position 0, at PTC) at the onset of SecA binding. The residues to the left of the dashed line are exposed outside the ribosomal tunnel exit. **b,c,** Representative SecA interaction profile of an IMP with a large periplasmic loop (**b**) and a secretory protein (**c**) that showed persistent SecA association upon CCCP treatment. Protein topology is shown above and colored as in Fig. 2b and Fig. 4c. Solid lines show the mean values and shaded areas show the differences between two independent biological replicates. **d,** Representative SecA interaction profile of an IMP with a large periplasmic loop that showed significantly reduced SecA association upon CCCP treatment. Protein topology is shown above and colored as in Fig. 2b. Solid lines show the mean values and shaded areas show the differences between two independent biological

replicates. **e,f,** Representative ribosome localization profiles of IMPs with a large periplasmic loop that showed significantly reduced SecA association upon CCCP treatment. Corresponding SecA interaction profiles shown in Fig. 6d and Supplementary Fig. 8d. Protein topology is shown above and colored as in Fig. 2b. Solid lines show the mean values and shaded areas show the differences between two independent biological replicates. **g,** Metagene ribosome localization profile of IMPs with no periplasmic loops larger than 45 amino acids, aligned to the N-terminus of their first TMD, in untreated and CCCP-treated cells. Solid lines show the mean values and shaded areas show the 95% CI. **h,** Representative ribosome localization profiles of an IMP without a large periplasmic loop in untreated and CCCP-treated cells. Corresponding SecA interaction profile shown in Fig. 6f. Protein topology is shown above and colored as in Fig. 2b. Solid lines show the mean values and shaded areas show the differences between two independent biological replicates.

Supplementary Table 1 Coverage of datasets generated in this study

Sample name	Uniquely aligned reads	Reads aligned to CDS
WT SecA interactome, rep1	34804789	30825565
WT SecA interactome, rep2	21788144	18970768
WT total translatome, rep1	38264052	32904519
WT total translatome, rep2	21240554	18188618
Δ*tig* SecA interactome, rep1	18356171	16289432
Δ*tig* SecA interactome, rep2	13406407	11886252
Δ*tig* total translatome, rep1	16774681	14282487
Δ*tig* total translatome, rep2	18691996	15850378
*secA*Δ*ZnBD* SecA interactome, rep1	31926390	28064636
*secA*Δ*ZnBD* SecA interactome, rep2	19381780	16553781
*secA*Δ*ZnBD* total translatome, rep1	42745477	36632557
*secA*Δ*ZnBD* total translatome, rep2	19980672	17155952
Δ*secB* SecA interactome, rep1	13629065	11996830
Δ*secB* SecA interactome, rep2	12963717	11397043
Δ*secB* total translatome, rep1	15436331	13228815
Δ*secB* total translatome, rep2	13572397	11655639
WT SecA interactome, rep1, CCCP	9399204	8240630
WT SecA interactome, rep2, CCCP	15525893	13483727
WT total translatome, rep1, CCCP	21846422	18660959
WT total translatome, rep2, CCCP	11769273	10012956
WT cytosolic monosomes, rep1	23024694	19551026
WT cytosolic monosomes, rep2	11718022	9951046
WT membrane monosomes, rep1	11362514	9916473
WT membrane monosomes, rep2	7295535	6361487
WT cytosolic monosomes, rep1, CCCP	17337810	15148035
WT cytosolic monosomes, rep2, CCCP	14996202	13067180
WT membrane monosomes, rep1, CCCP	25583593	23464722
WT membrane monosomes, rep2, CCCP	22731118	20803452
