# Supplementary material for: Ribosome profiling reveals multiple roles of SecA in cotranslational protein export
Source: Nat Commun. 2022 Jun 13;13:3393. doi: 10.1038/s41467-022-31061-5 (PMC9192764; doi:10.1038/s41467-022-31061-5)
Supplement: Supplementary file 2 — Description of Additional Supplementary Files [file 41467_2022_31061_MOESM2_ESM.pdf]

Description of Additional Supplementary Files

File Name: Supplementary Data 1
Description: Selective ribosome profiling data analysis. Membrane protein topology: S, signal sequence; M, transmembrane domain; I, cytoplasmic loop; o, periplasmic loop.
