# Supplementary material for: Ribosome profiling reveals multiple roles of SecA in cotranslational protein export
Source: Nat Commun. 2022 Jun 13;13:3393. doi: 10.1038/s41467-022-31061-5 (PMC9192764; doi:10.1038/s41467-022-31061-5)
Supplement: Supplementary file 4 — Reporting Summary [file 41467_2022_31061_MOESM4_ESM.pdf]

nature portfolio

Corresponding author(s): Shu-ou Shan

Last updated by author(s): May 16, 2022

Reporting Summary

Nature Portfolio wishes to improve the reproducibility of the work that we publish. This form provides structure for consistency and transparency in reporting. For further information on Nature Portfolio policies, see our Editorial Policies and the Editorial Policy Checklist.

Statistics

For all statistical analyses, confirm that the following items are present in the figure legend, table legend, main text, or Methods section.

n/a	Confirmed	
☐	☒	The exact sample size (*n*) for each experimental group/condition, given as a discrete number and unit of measurement
☐	☒	A statement on whether measurements were taken from distinct samples or whether the same sample was measured repeatedly
☐	☒	The statistical test(s) used AND whether they are one- or two-sided<br>*Only common tests should be described solely by name; describe more complex techniques in the Methods section.*
☒	☐	A description of all covariates tested
☐	☒	A description of any assumptions or corrections, such as tests of normality and adjustment for multiple comparisons
☐	☒	A full description of the statistical parameters including central tendency (e.g. means) or other basic estimates (e.g. regression coefficient) AND variation (e.g. standard deviation) or associated estimates of uncertainty (e.g. confidence intervals)
☐	☒	For null hypothesis testing, the test statistic (e.g. *F*, *t*, *r*) with confidence intervals, effect sizes, degrees of freedom and *P* value noted<br>*Give P values as exact values whenever suitable.*
☒	☐	For Bayesian analysis, information on the choice of priors and Markov chain Monte Carlo settings
☒	☐	For hierarchical and complex designs, identification of the appropriate level for tests and full reporting of outcomes
☐	☒	Estimates of effect sizes (e.g. Cohen's *d*, Pearson's *r*), indicating how they were calculated

Our web collection on statistics for biologists contains articles on many of the points above.

Software and code

Policy information about availability of computer code

Data collection	Sequencing data was collected on Illumina Hiseq 2500 by the Millard and Muriel Jacobs Genetics and Genomics Laboratory at California Institute of Technology
Data analysis	Cutadapt v3.5, Bowtie v1.3, python v 3.7, ImgaeJ v1.52, WebLogo3, TOPCONS2, TMHMM2.0, SignalP-5.0

For manuscripts utilizing custom algorithms or software that are central to the research but not yet described in published literature, software must be made available to editors and reviewers. We strongly encourage code deposition in a community repository (e.g. GitHub). See the Nature Portfolio guidelines for submitting code & software for further information.

Data

Policy information about availability of data

All manuscripts must include a data availability statement. This statement should provide the following information, where applicable:

- Accession codes, unique identifiers, or web links for publicly available datasets
- A description of any restrictions on data availability
- For clinical datasets or third party data, please ensure that the statement adheres to our policy

The accession number for the data reported in this paper is GEO:GSE185572. The protein structures used to calculate absolute contact order were downloaded from AlphaFold Protein Structure Database (https://alphafold.ebi.ac.uk/). Source data are provided with this paper in Supplementary Data 1 and Source Data file.

Field-specific reporting

Please select the one below that is the best fit for your research. If you are not sure, read the appropriate sections before making your selection.

☒ Life sciences ☐ Behavioural & social sciences ☐ Ecological, evolutionary & environmental sciences

For a reference copy of the document with all sections, see nature.com/documents/nr-reporting-summary-flat.pdf

Life sciences study design

All studies must disclose on these points even when the disclosure is negative.

Sample size	No statistical methods were used to predetermine the sample size. All ribosome profiling experiments were performed in 2-3 biological replicates. High correlation between the replicates is observed and considered to be sufficient.
Data exclusions	When calculating the gene-level enrichment, the raw reads and RPM-normalized reads at each codon for every gene were summed after excluding the first five and last five codons to avoid known ribosome profiling biases. Only the genes with more than 100 reads in both biological replicates of translatome and SecA interactome were included. For metagene analyses of SecA enrichment, the first five and last five codons were excluded, and only the genes with an average reads per codon of > 0.5 in both the translatome and SecA interactome were used.
Replication	All ribosome profiling was performed in 2-3 replicates and are reproducible as assessed by Pearsons's correlation coefficient. All western blots were repeated twice on two independently grown cultures for each condition, and the replications are reproducible .
Randomization	Randomization is not applicable to this study, as there was no allocation into subgroups.
Blinding	Blinding is not applicable to this study, as there was no subgroup allocation, and the study does not include animals and/or human research participants.

Reporting for specific materials, systems and methods

We require information from authors about some types of materials, experimental systems and methods used in many studies. Here, indicate whether each material, system or method listed is relevant to your study. If you are not sure if a list item applies to your research, read the appropriate section before selecting a response.

Materials & experimental systems

n/a	Involved in the study	
☐	☒	Antibodies
☒	☐	Eukaryotic cell lines
☒	☐	Palaeontology and archaeology
☒	☐	Animals and other organisms
☒	☐	Human research participants
☒	☐	Clinical data
☒	☐	Dual use research of concern

Methods

n/a	Involved in the study	
☒	☐	ChIP-seq
☒	☐	Flow cytometry
☒	☐	MRI-based neuroimaging

Antibodies

Antibodies used	Rabbit anti-SecA antibody (a gift from Tom A. Rapoport, used at 1:1000 dilution)<br>Mouse anti-S13 antibody (DSHB 193E11E5B11, used at 1:3000 dilution)<br>Rabbit anti-YidC antibody (a gift from Ross E. Dalbey, used at 1:5000 dilution)<br>Mouse anti-DnaK antibody (Abcam ab69617, used at 1:3000 dilution)<br>IRDye® 800CW Goat anti-Mouse IgG Secondary Antibody (LI-COR 926-32210, used at 1:15000 dilution)<br>IRDye® 800CW Goat anti-Mouse IgG Secondary Antibody (LI-COR 926-32211, used at 1:15000 dilution)
Validation	Rabbit anti-SecA antibody (a gift from Tom A. Rapoport). Application tested in the manuscript: WB(1:1000) in E.coli.<br>Mouse anti-S13 antibody (DSHB 193E11E5B11). Company website application listed: WB, IF, IHC and ICC in E.coli. Application tested in the manuscript: WB(1:5000) in E.coli.<br>Rabbit anti-YidC antibody (a gift from Ross E. Dalbey). Application tested in the manuscript: WB(1:5000) in E.coli.<br>Mouse anti-DnaK antibody (Abcam ab69617). Company website application listed: WB in E.coli. Application tested in the manuscript: WB(1:3000) in E.coli.
